# STING controls opioid-induced itch and chronic itch via spinal tank-binding kinase 1-dependent type I interferon response in mice

**DOI:** 10.1186/s12974-023-02783-0

**Published:** 2023-04-30

**Authors:** Nan Li, Chunyan Wang, Yuying Zhao, Yigang Wang, Tianyu Gao, Yonghao Yu, Guolin Wang, Linlin Zhang

**Affiliations:** 1 Department of Anesthesiology, Tianjin Medical University General Hospital, Tianjin, 300052 China; 2 Tianjin Research Institute of Anesthesiology, Tianjin, 300052 China

**Keywords:** STING, Opioid-induced itch, Chronic itch, TBK1, Type I interferon

## Abstract

**Background:**

Patients receiving epidural or intrathecal opioids administration for neuraxial analgesia frequently suffer from an irritating itch. STING (stimulator of interferon genes), an innate immune modulator, is strongly implicated in pain pathogenesis via neuron-immune modulation. Given that pain and itch share some common neurocircuits, we evaluate the therapeutic potential of STING agonists in opioid-induced itch and chronic itch.

**Methods:**

Opioids (morphine, fentanyl and sufentanil) were intrathecally injected to induce acute itch. Chronic itch was induced by dry skin and contact dermatitis. Opioids analgesic effect, itch-induced scratching behavior, spinal expression of STING, phosphorylation of TBK1 (tank-binding kinase 1), IRF3 (interferon regulatory factor-3) and ERK (extracellular signal-regulated kinase), as well as production of IFN-α and IFN-β were examined. STING agonists (DMXAA and ADU-S100), TBK1 inhibitor, recombinant IFN-α and IFN-β elucidated the mechanism and treatment of itch. Whole-brain functional connectivity was evaluated using resting-state fMRI.

**Results:**

We report the primary expression of STING protein by the spinal dorsal horn neurons. Intraperitoneal injection of DMXAA dose-dependently reduces morphine-induced scratch bouts, without impairing morphine antinociception. Simultaneously, DMXAA alleviates fentanyl- and sufentanil-induced itching-like behavior, and chronic scratching behavior caused by dry skin and contact dermatitis. Furthermore, DMXAA drastically increases spinal phosphorylation of TBK1 and IRF3 following morphine exposure, dry skin and contact dermatitis. DMXAA-induced anti-pruritus effects and spinal productions of IFN-α and IFN-β are compensated by intrathecal delivery of the TBK1 inhibitor. Also, ADU-S100, recombinant IFN-α and IFN-β exhibits remarkable attenuation in scratching behaviors after morphine injection and dermatitis. Recombinant IFN-α inhibits morphine-induced spinal phosphorylation of ERK. Finally, DMXAA prevents dermatitis-induced the increase of cerebral functional connectivity between regions of interests such as primary somatosensory cortex, piriform cortex, retrosplenial cortex, colliculus and ventral thalamus.

**Conclusions:**

STING activation confers protection against opioid-induced itch and chronic itch through spinal up-regulation of TBK1-IRF3-type I interferon cascades in mice, suggesting that STING agonists are promising candidates in translational development for pruritus relief.

**Supplementary Information:**

The online version contains supplementary material available at 10.1186/s12974-023-02783-0.

## Introduction

Opioids are the current mainstay for clinical pain management but have several side effects such as hyperalgesia, nausea, respiratory depression following systemic administration [1, 2]. Epidural or spinal opioids are frequently added to local anesthetics for neuraxial analgesia in labor, perioperative care and chronic pain conditions [3–5]. Pruritus is one of the most common and irritating side effects of intrathecal opioid application, which impairing patient satisfaction with analgesia and anesthesia [6, 7]. Furthermore, patients undergoing chronic itch after dry skin and contact dermatitis usually experience touch-elicited itch (alloknesis) and spontaneous itch, for which is often refractory to most current therapies, thus emphasizing the need for improved therapeutic medications [8, 9]. Accumulating evidence emphasizes that neuroinflammation and central pruriceptive sensitization drive spinal perception of itch [10–13]. Yet, the specific mechanisms underlying opioid-induced pruritus and chronic pruritus remain unclear.

Pain and itch, transmitted by nociceptors and pruriceptors, facilitate host defense by organisms alerts following the recognition of potentially noxious stimuli, such as tissue injuries and pathogens [14]. Stimulator of interferon genes (STING), a critical sensor of self- and pathogen-derived DNA, is identified as a key signaling molecule for the elimination of pathogens and damaged host cells during innate and adaptive immunity [15]. The production of type I interferon (IFN-I), including IFN-α and IFN-β, is a cardinal feature of STING activation via initiating and sustaining the phosphorylation of tank-binding kinase 1 (TBK1) and interferon regulatory factor 3 (IRF3) [16]. More importantly, STING activation in the central nervous system is effective against different neurological disorders through TBK1-IRF3-dependent IFN-I response and regulation of neuroinflammation [17–20]. Recent reports highlight that STING controls nociception in animals with pathological pain via neuron-immune modulation [21, 22]. Given that pain and pruritus share some striking similarities, we investigated whether STING agonism could suppress neuraxial opioid-induced itch and chronic itch. Additionally, canonical IFN-I signaling is gradually recognized as the therapeutic targets for acute and chronic pain following surgeries, infections and injuries [23, 24]. However, little is known about the interaction between STING and TBK1-IRF3-IFN-I cascades in itch neurocircuits.

In this study, we characterized the potential role of intraperitoneal (i.p.) the murine STING agonist DMXAA and the cross-species STING agonist ADU-S100 in pruritus using acute itch models induced by opioids (morphine, fentanyl, sufentanil), and pruritogens (compound 48/80, chloroquine), and chronic itch models induced by dry skin and contact dermatitis. Also, spinal phosphorylation of TBK1 and IRF3 was evaluated, spinal production of IFN-α and IFN-β was measured, and a selective TBK1 inhibitor (BX795), IFN-I neutralizing antibodies and recombinant IFN-I were utilized to validate the anti-pruriceptive mechanisms of STING agonists in these mouse models. As the conscious experience of pruritus is ultimately generated in the brain, assessing anti-itch effects of STING agonism on the brain in a chronic itch state might give further insights into how effective itch relief is. Thus, we also evaluated whole-brain functional connectivity using resting-state functional magnetic resonance imaging (fMRI) in contact dermatitis-induced chronic itch.

## Methods

### Animals

Adult C57BL/6 J mice (males and females, 8–10 weeks old) were raised in an artificially regulated 12-h light–dark environment with free access to food and water. All animals were purchased from the experimental animal center of the Chinese Academy of Military Medical Science. All experimental studies and protocols were conducted in strict accordance with the National Institutes of Health Guide for the Care and Use of Laboratory Animals, and approved by the Animal Ethical and Welfare Committee of Tianjin Medical University (Tianjin, China). The investigators blinded to the treatments collected the behavioral data. Animals were habituated to the testing environment daily for at least two days before any baseline testing. The room temperature and humidity remained stable for all experiments.

### Drug and administration

Morphine was purchased from Northeast Pharmaceutical Group Shenyang First Pharmaceutical Co. LTD (Shenyang, Liaoning, China). Fentanyl and sufentanil were purchased from RenFu Pharmaceutical Co. (Yichang, Hubei, China). The compound 48/80, chloroquine (CQ) and BX795 were from Sigma-Aldrich (St. Louis, MO, USA). Diphenylcyclopropenone (DCP) was from Shanghai Aladdin Biochem Technology Co., Ltd (Shanghai, China). DMXAA and ADU-S100 were from MedChemExpress (Shanghai, China). Recombinant IFN-α, recombinant IFN-β, IFN-α neutralizing antibody (anti-IFN-α) and IFN-β neutralizing antibody (anti-IFN-β) were from PBL Assay Science (Piscataway, NJ, USA). For intrathecal (i.t.) injection, spinal cord puncture was made with a 30 G needle between the L_4_ and L_5_ level to deliver drugs (5 µl) to the cerebral spinal fluid [21].

### Mouse model of acute itch

On the day of behavioral testing, mice were individually placed in small plastic chambers (15 × 15 × 15 cm^3^) on an elevated metal mesh floor and allowed at least 30 min for habituation. Mice were given i.t. injections of morphine (0.3 nmol), fentanyl (0.5 μg) and sufentanil (0.5 μg), or intradermal (i.d.) injections of 50 μl of compound 48/80 (100 μg) or CQ (200 μg) via a 30G needle into the nape of the neck [10, 11]. After the injection, scratching behavior was quantified for 30 min. A scratch was counted when the mouse lifted its hind paw to scratch and returned the paw to the floor [11].

### Dry-skin induced chronic itch

The hair of the nape was shaved 2 days before intervention. Dry skin was induced by application of a 1.5 ml 1:1 mixture of acetone and diethyl ether for 10 min, followed by clean water for 30 s (AEW) twice a day (morning and evening) for 8 days [10]. The spontaneous scratching behavior was recorded for 1 h. Bouts of scratching were then counted.

### DCP induced chronic itch

Diphenylcyclopropenone (DCP) is a topical immunotherapy agent for treating alopecia areata, which frequently causes severe adverse effects including eczematous skin, contact dermatitis as well as intense scratching behaviors [25]. Thus, we employed DCP to induce contact dermatitis and chronic itch. Specifically, DCP (1% in acetone, 0.2 ml) was painted onto neck skin after the hair of the nape was shaved. After one week, we challenged the animals with painting the nape skin with 0.2 ml 0.5% DCP, which was applied daily for 10 days [10]. The spontaneous scratching behavior was recorded for 1 h after each DCP application. Bouts of scratching were then counted.

### Morphine analgesia analysis

Tail-flick test was conducted as previously described [11]. Briefly, mice were gently held by hand with a terry glove with tail exposed. The distal 3 cm end of the tail was immersed into a 50 °C hot water bath. The tail-flick latency was recorded as the time required for the animals to flick or remove its tail from the water, with a maximum cut-off value of 15 s to prevent heat damage.

### Rotarod test

Mice were trained on the rotarod for 3 min at a speed of 10 rpm, until they no longer fell off. For testing, the speed of rotation was set at 10 rpm for 60 s and subsequently accelerated to 80 rpm in 5 min. The falling latency after the starting of the acceleration was recorded [10].

### Western blot

Animals were sacrificed under deep isoflurane anesthesia. The dorsal horn of spinal cord segments was removed rapidly and snap-frozen in liquid nitrogen. Samples were mechanically homogenized in ice-cold radioimmune precipitation assay buffer containing phenylmethanesulfonyl fluoride (Abcam, Cambridge, UK). The protein content was determined using the bicinchoninic acid assay method. The loading and blotting of an equivalent number of total proteins were verified using a membrane with monoclonal mouse anti-β-actin antibody (1:5000; Sigma-Aldrich). The samples were resolved on a 10% SDS-PAGE gel, transferred to nitrocellulose membrane, and probed with polyclonal rabbit antibodies against anti-STING (1:1000, Abcam), TBK1 (1:1000, Cell Signalling Technology, Danvers, USA), p-TBK1 at Ser-172 (1:1000, Cell Signalling Technology), IRF3 (1:1000, Cell Signalling Technology), p-IRF3 at Ser-396 (1:1000, Cell Signalling Technology), p-ERK (1:1000, Cell Signalling Technology), followed by incubation with horseradish peroxidase-conjugated secondary antibodies (1:2000, Jackson Immuno Research). The membrane-bound secondary antibodies were visualized with enhanced chemiluminescence (Thermo Scientific, Rockford, IL, USA) and quantified with Image-Pro Plus software (Media Cybernetics Inc).

### ELISA analysis

An enzyme-linked immunosorbent assay (ELISA) was used to measure the concentrations of IFN-α and IFN-β in the spinal cord. Mouse high-sensitivity IFN-α ELISA kit and IFN-β ELISA kit were purchased from PBL Assay Science. Spinal tissues were homogenized in a lysis buffer containing protease and phosphatase inhibitors. Tissue samples were centrifuged at 12,500 ×*g* for 10 min and the supernatant was collected. BCA Protein Assay (Pierce) was employed to determine protein concentrations. For each reaction in a 96-well plate, 100 μg of proteins of samples were used. All ELISA experiments followed the manufacturer’s protocol. The optical densities of samples were measured using an ELISA plate reader (Bio-Rad) and the levels of IFN-α and IFN-β were calculated using the standard curves and ELISA results were reported normalized to protein concentration (pg/mg spinal tissue).

### Immunohistochemistry

Animals were deeply anesthetized with isoflurane and perfused through the ascending aorta with PBS (phosphate-buffered saline), followed by 4% paraformaldehyde with 0.1% picric acid in 0.16 M phosphate buffer. Spinal cord (30 μm, free-floating) was cut in a cryostat. The sections were first blocked with 1% BSA + 0.1% Triton X 100 for 1 h at room temperature, and then incubated overnight at 4 °C with the primary antibodies. The following primary antibodies were used: anti-STING (1:100, Abcam), anti-NeuN (1:200, Abcam), anti-GFAP (1:200, Cell Signaling Technology), and anti-IBA-1 (1:200, Abcam). After rinsing three times with PBS, the sections were incubated with fluorescence-labeled secondary antibody for 1 h. Images were collected using a fluorescence microscope (Olympus, Japan).

### Resting-state fMRI

For examining the whole-brain resting state-functional connectivity (FC) differences, we used the reference atlas of the Allen Institute for Brain Sciences (AIBS), consisting of 75 regions of interests (ROIs). Resting-state fMRI data were used to explore the cerebral cortical regions. MRI data were collected using a small animal 9.4 T Biospec 94/30 preclinical system (Bruker, Ettlingen, Germany), equipped with a gradient coil with a 12-cm inner diameter, a maximum gradient strength of 660 mT/m, and a 4-channel animal head coil.

For MRI data acquisition, animals were initially anesthetized with 3–5% isoflurane in an oxygen-rich environment and given an intraperitoneal bolus injection of dexmedetomidine at a dose of 0.03 mg/kg. Then, they were placed prone with their head fixed with a tooth bar and ear bars. Their physiological conditions, including respiration rate, heart rate and pulse oxygen saturation, were monitored (SA Instruments, Stony Brook, NY, USA), and their core body temperature was controlled to 37 ± 0.5 °C using a controlled warm water system (Thermo Scientific SC100, Waltham, MA, USA). During MRI scanning, dexmedetomidine was continuously infused intraperitoneally at a dose of 0.015 mg/kg/h and isoflurane was reduced to 0.5–0.75%. Functional data was acquired using a gradient-echo echo planar imaging (GE-EPI) sequence sensitive to the blood-oxygen level dependent (BOLD) contrast with the following parameters: Field of view (FOV) = 16 mm x 8 mm, slice thickness (SLTH) = 0.5 mm, repetition time (TR) = 1000 ms, echo time (TE) = 12 ms, Flip angle = 25°, 15 contiguous slices. The fMRI data were preprocessed by SPM8 (www.fil.ion.ucl.ac.uk/spm), MATLAB (Version 2015b, MathWorks, USA), and data processing and analysis of brain imaging (DPABI) V4.3. Briefly, fMRI data were converted to NIFTI format, and the voxel size was scaled up by a factor of 10 to fit standard neuroimaging software packages designed for human brain imaging. After removing the first 10 volumes, slice timing and realignment were performed by DPABI. The realigned fMRI images were co-registered to their corresponding T2-weighted images and were then normalized to mouse brain template in standard space. Finally, spatial smoothing (FWHM = 6 mm), regression of motion parameters and the signals of white matter and cerebrospinal fluid, and bandpass filtering (0.01–0.1 Hz) were carried out by DPABI.

Connectivity matrices for each subject were calculated as the Fisher transformed correlation between the hemodynamic response function-weighted regional time series of these 75 ROIs. Then, we tested whether there were any pairs of connections that showed significantly different mean connectivity among the three groups. The statistical analysis for FC was performed using code written in MATLAB.

### Quantification and statistics

Sample sizes were estimated on the basis of previous studies for similar types of behavioral and biochemical analyses [11, 21]. Animals were randomly assigned to different experimental groups. Both males and females were included in each group in a sex-matched manner. The data from both sexes were combined and used equally throughout this study, as no sex differences were observed in key behavioral experiments (Additional file 1: Fig. S1, Additional file 2: Fig. S2, Additional file 3: Fig. S3). All data were expressed as mean ± SD (standard deviation), and no data were excluded for observation and statistics. Differences between groups were compared using two-tailed Student’s *t* test, one-way or two-way ANOVA followed by Bonferroni *post-hoc* test. The criterion for statistical significance was *P* < 0.05.

## Results

### DMXAA attenuates intrathecal opioid-induced acute itch, without impairing opioid analgesia

**Fig. 1:**
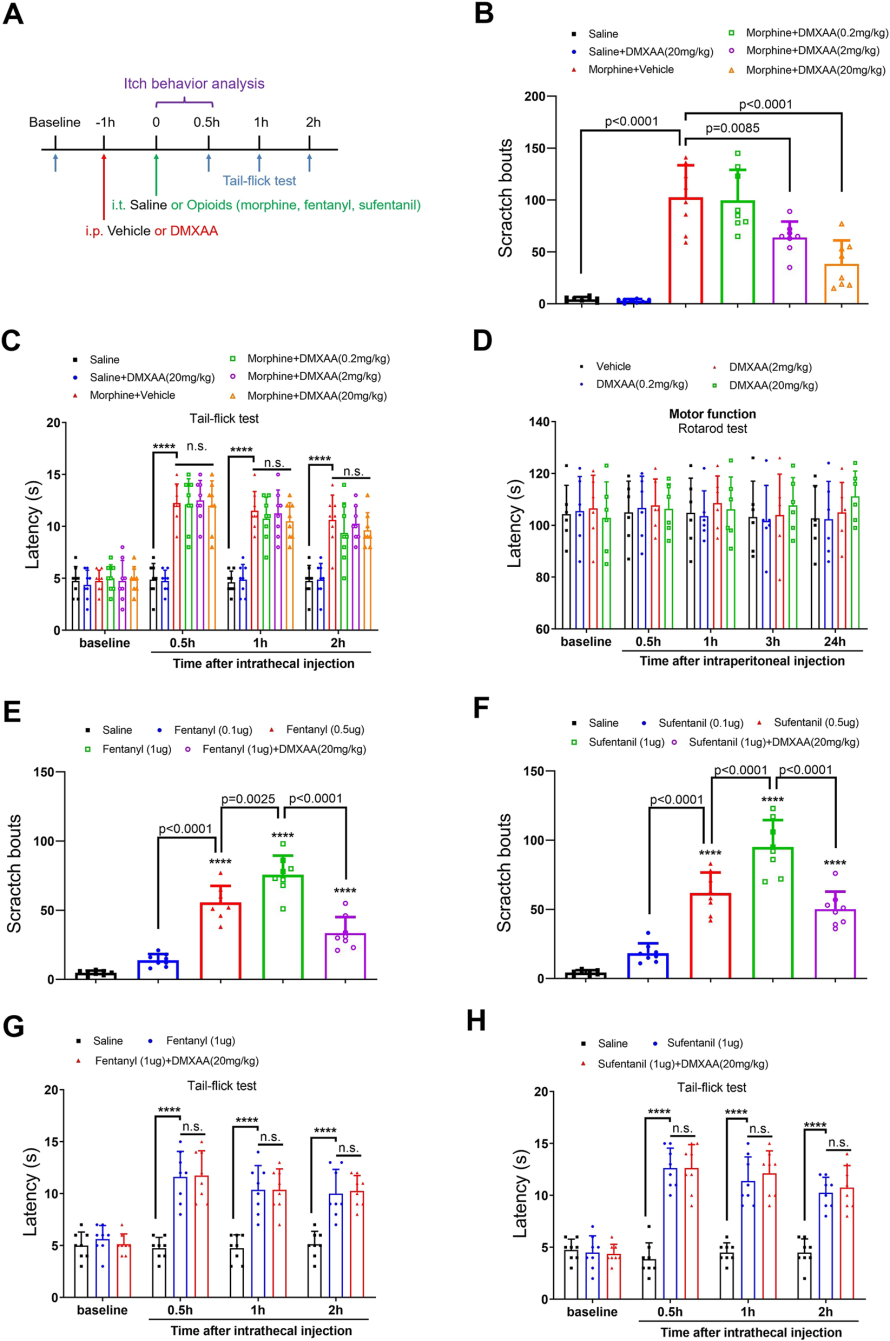
The murine STING agonist DMXAA reduces intrathecal opioid-induced pruritus, without compromising opioids antinociception in mice. **A** Experimental design to test the anti-pruritus effect of DMXAA in the mouse model of intrathecal (i.t.) opioid-induced itch. **B** Intraperitoneal (i.p.) pre-administration of DMXAA attenuates i.t. morphine-induced scratching behaviors in a dose-dependent manner. n = 8 mice/group. **C** Morphine antinociception, assessed by Tail-flick test, is not impaired by DMXAA therapy. n = 8 mice/group. **D** Locomotor function, assessed by Rotarod test, is not impaired by DMXAA therapy. n = 6 mice/group. **E** Fentanyl- and **F** sufentanil-induced scratching behavior is alleviated by DMXAA treatment. n = 8 mice/group. **G** Fentanyl- and **H** sufentanil-induced analgesia is unaffected by DMXAA treatment. n = 8 mice/group. Data are expressed as mean ± SD and analyzed by one-way ANOVA with Bonferroni post hoc test (**B**, **E**, **F**), and two-way ANOVA with Bonferroni post hoc test (**C**, **D**, **G**, **H**). Compared with group Saline, ^****^P < 0.0001. n.s., not significant

To investigate a specific role of STING agonism in opioid-induced itch, the model of intrathecal morphine (0.3 nmol) injection was employed and the murine STING agonist DMXAA (i.p., 0.2, 2 and 20 mg/kg) was administered 60 min prior to morphine exposure (Fig. 1A). First, we found the robust scratching behaviors and the remarkable increase of tail-flick latency following morphine injection, suggesting that morphine (i.t., 0.3 nmol) is sufficient to provide adequate antinociception and induce itch phenotype (Fig. 1B and C). Intriguingly, DMXAA at 2 and 20 mg/kg but not 0.2 mg/kg reduced i.t. morphine-induced pruritus, as characterized by the abrupt decrease in scratch bouts (F (5, 42) = 36.68, *P* < 0.0001, one-way ANOVA, Fig. 1B). By contrast, DMXAA failed to affect morphine-caused increase of tail-flick latency (Fig. 1C). Moreover, DMXAA did not change locomotor function in vehicle-treated mice (Fig. 1D), suggesting that the prevention of pruritus by DMXAA was not due to a defect of locomotor activity. Also, we observed no sex differences in i.t. morphine-induced itching-like behaviors and anti-pruritus effects of DMXAA between male and female mice (F (1, 60) = 0.099, *P* = 0.7541, two-way ANOVA, Additional file 1: Fig. S1A).

Although many studies have explored the prevention of neuraxial morphine-induced pruritus in animals, relatively few have focused on lipophilic opioids, such as fentanyl and sufentanil. A meta-analysis of 24 clinical trials reported that the incidence of pruritus associated with neuraxial fentanyl and sufentanil is as high as that with neuraxial morphine [26]. Thus, we sought to evaluate whether STING activation could inhibit intrathecal lipophilic opioid-evoked itch. Noteworthy, we found that i.t. fentanyl and sufentanil administration caused the scratching behavior in a dose-dependent manner, moreover, both fentanyl (1 μg) and sufentanil (1 μg) generated most evident scratching bouts, which was effectively reduced by DMXAA (i.p., 20 mg/kg) pre-treatment (F (4, 35) = 70.48, *P* < 0.0001, and F (4, 35) = 64.01, *P* < 0.0001, respectively, one-way ANOVA, Fig. 1E and F). Whereas optimal analgesia by intrathecal lipophilic opioids injection was not compromised (Fig. 1G and H). In addition, the anti-pruritus effects of DMXAA on neuraxial fentanyl/sufentanil-induced itching-like behaviors were indistinguishable between sexes (F (1, 30) = 0.366, *P* = 0.5496, and F (1, 30) = 0.116, *P* = 0.7363, respectively, two-way ANOVA, Additional file 1: Fig. S1B and C).

### DMXAA increases spinal phosphorylation of TBK1 and IRF3 in mice after intrathecal morphine administration

**Fig. 2:**
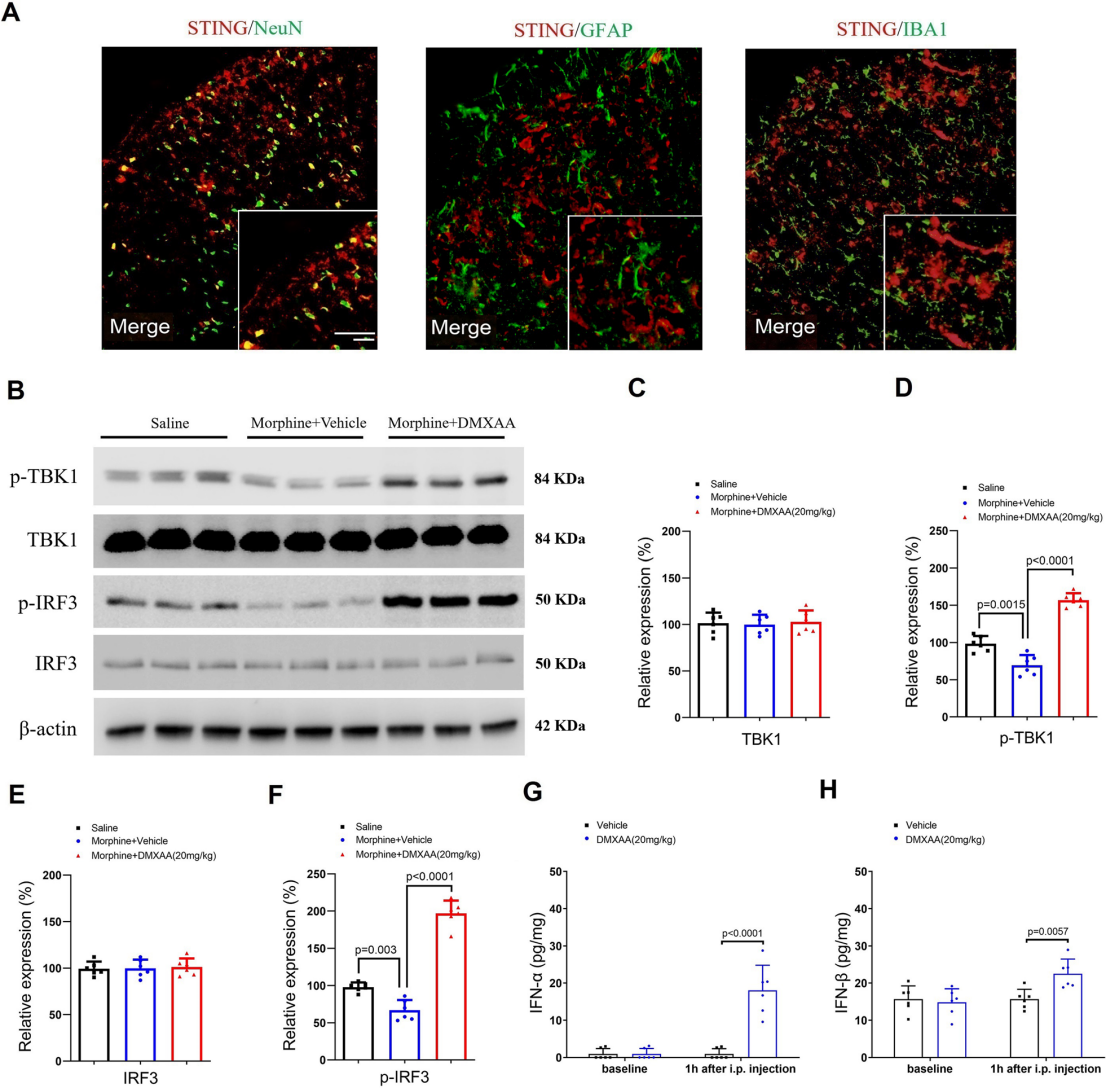
STING agonism elevates neuraxial morphine-induced spinal decrease of TBK1 and IRF3 phosphoration. **A** Double staining of STING (red) with markers (green) of different cells in the spinal dorsal horn—neuron (NeuN), astrocyte (GFAP), and microglia (IBA-1). Scale bar, 100 μm. **B**-**F** STING agonist DMXAA (i.p., 20 mg/kg) was administered 60 min prior to morphine (i.t., 0.3 nmol) exposure. Western blot showed that DMXAA treatment affects spinal TBK1 and IRF3 phosphorylation but not expression at 30 min after morphine injection. **G** and **H** Spinal concentrations of IFN-α and IFN-β at 1 h after DMXAA treatment were measured using ELISA assay. n = 6 mice/group. Data are expressed as mean ± SD and analyzed by one-way ANOVA with Bonferroni post hoc test (**C**–**F**), and two-way ANOVA with Bonferroni post hoc test (**G** and **H**)

Double staining revealed that STING protein highly co-localized with a neuronal marker NeuN but not astrocytic marker GFAP and microglial marker IBA-1 (Fig. 2A), thus manifesting the primary expression of STING protein by the spinal dorsal horn neurons and suggesting that alleviation of morphine-induced itch by STING agonism may be via rapid modulation of neuronal activity. In general, STING activation subsequently recruits TBK1 to facilitate the phosphorylation of TBK1 and IRF3, which ultimately causes to the production of IFN-I [16]. The canonical STING-dependent IFN-I cascade has emerged as a therapeutic target for acute and chronic pain control [21–24]. We next investigated these key signaling proteins in the dorsal horn by Western blot. We found the significant decrease of TBK1 and IRF3 phosphorylation at 30 min after spinal exposure to morphine while the expression of TBK1 and IRF3 proteins was not changed (Fig. 2B–F). Strikingly, pre-administration of DMXAA (i.p., 20 mg/kg) prevented the down-regulation of TBK1 phosphorylation by neuraxial morphine (F (2, 15) = 92.87, *P* < 0.0001, one-way ANOVA, Fig. 2B and D). In parallel, spinal phosphorylation of IRF3 in mice with morphine exposure was elevated by systemic DMXAA therapy (F (2, 15) = 157.3, *P* < 0.0001, one-way ANOVA, Fig. 2B and F). Furthermore, spinal expressions of IFN-α and IFN-β were induced at 1 h after DMXAA administration (F (1, 20) = 33.8, *P* < 0.0001, and F (1, 20) = 4.384, *P* = 0.0492, respectively, two-way ANOVA, Fig. 2G and H). These detailed results suggest that STING activation may control opioid-induced itch via regulating spinal TBK1-IRF3-IFN-I response.

### DMXAA attenuates dry skin-induced chronic itch, but not pruritogens-induced acute itch

**Fig. 3:**
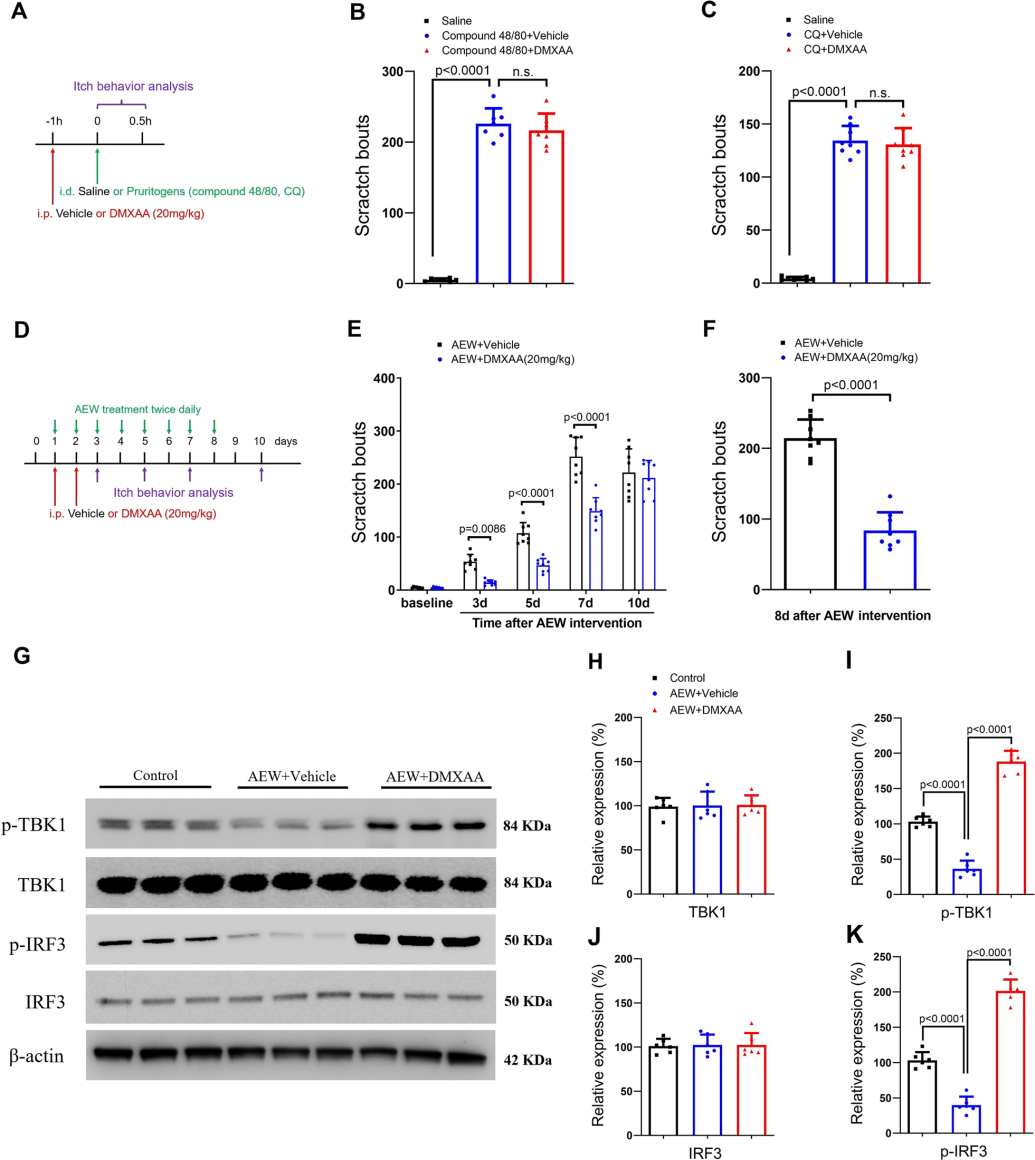
The murine STING agonist DMXAA reduces dry skin-induce chronic itch but not pruritogens-induced acute itch in mice. **A** Experimental design to test the anti-pruritus effect of DMXAA in the mouse model of intradermal (i.d.) injections of pruritogens (compound 48/80 and CQ)-induced acute pruritus. **B** and **C** DMXAA does not affect pruritogens-induced scratching behaviors. n = 8 mice/group. **D** Experimental design to test the anti-pruritus effect of DMXAA on AEW-induced chronic pruritus in dry skin model. **E** pre-treatment with DMXAA reduces AEW-induced scratching behaviors. n = 8 mice/group. **F** Single application of DMXAA on days 8 after AEW exposure reduced the established pruritus. n = 8 mice/group. **G**–**K** Western blot showed that two injections of DMXAA (i.p., 20 mg/kg) daily from day 1 to 2 after AEW exposure affect spinal TBK1 and IRF3 phosphorylation (but not expression) on day 3 after AEW intervention. n = 6 mice/group. Data are expressed as mean ± SD and analyzed by one-way ANOVA with Bonferroni post hoc test (**B**, **C**, **H**–**K**), unpaired two-tailed *t* test (**F**), and two-way ANOVA with Bonferroni post hoc test (**E**). n.s., not significant

We then examined the potential contribution of STING activation to itch in different mouse models of pruritus. It is well indicated that pruritogens-induced acute itch is often characterized as histaminergic itch caused by compound 48/80, and non-histaminergic itch provoked by CQ [27, 28]. Herein, compound 48/80-induced scratch bouts and CQ-induced pruritus were not altered by DMXAA pre-administration (Fig. 3A–C), suggesting that STING is not implicated in histaminergic and non-histaminergic acute itch. AEW intervention elicits skin dehydration and initiates chronic pruritus. Mice received two injections of DMXAA (i.p., 20 mg/kg) daily from day 1 to 2 (in the early phase) after AEW treatment (Fig. 3D). We found that scratching behavior started on 3 days and lasted for at least 1 week following AEW exposure. Interestingly, systemic DMXAA injections effectively prevented AEW-induced chronic itch (F (1, 70) = 62.28, *P* < 0.0001, two-way ANOVA, Fig. 3E). A single delivery of DMXAA on 8 days (in the late phase) after AEW produced a rapid suppression of the established chronic itch (*P* < 0.0001, two-tailed Student’s *t* test, Fig. 3F). But we observed no sex differences in AEW-induced pruritus and anti-pruritus effects of DMXAA between both sexes (Additional file 2: Fig. S2). More strikingly, spinal down-regulation of TBK1 and IRF3 phosphorylation (but not expression) on day 3 after AEW exposure was significantly increased following DMXAA administration (F (2, 15) = 244.4, *P* < 0.0001, and F (2, 15) = 221.8, *P* < 0.0001, respectively, one-way ANOVA, Fig. 3G–K).

### DMXAA ameliorates chronic itch and increases the phosphorylation of TBK1 and IRF3 in mice with contact dermatitis

**Fig. 4:**
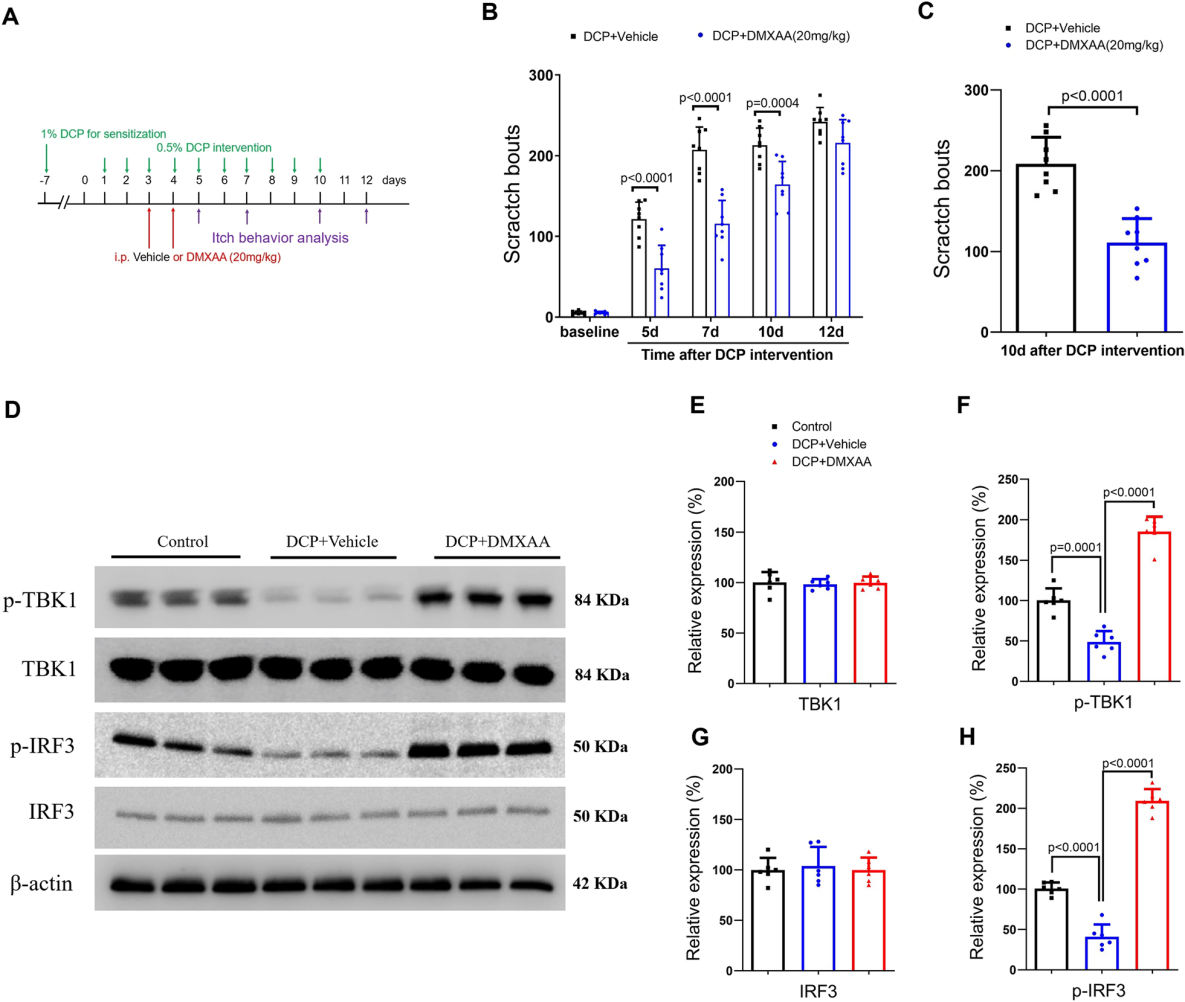
The murine STING agonist DMXAA elevates spinal phosphorylation of TBK1 and IRF3 in dermatitis-induce chronic pruritus. **A** Experimental design to test the anti-pruritus effect of DMXAA in contact dermatitis model. **B** pre-treatment with DMXAA reduces DCP-induced scratching behaviors. n = 8 mice/group. **C** Single application of DMXAA on days 10 after DCP exposure reduced the established pruritus. n = 8 mice/group. **D**–**H** Western blot showed that two injections of DMXAA (i.p., 20 mg/kg) daily from day 3 to 4 after DCP exposure affect spinal TBK1 and IRF3 phosphorylation (but not expression) on day 5 after DCP intervention. n = 6 mice/group. Data are expressed as mean ± SD and analyzed by two-way ANOVA with Bonferroni post hoc test (**B**), unpaired two-tailed *t* test (**C**), and one-way ANOVA with Bonferroni post hoc test (**E**–**H**)

To further examine the role of STING in chronic pruritus, mice were challenged with DCP to elicit and sustain contact dermatitis. First, mice received two injections of DMXAA (i.p., 20 mg/kg) daily from days 3 to 4 (in the early phase) after DCP treatment (Fig. 4A). We found that robust and persistent itch started on 5 days and continued for 1 week following DCP exposure, which was drastically reduced by systemic DMXAA therapy (F (1, 70) = 78.13, *P* < 0.0001, two-way ANOVA, Fig. 4B). Similarly, a single treatment of DMXAA on day 10 after DCP exposure significantly inhibited the established pruritus (*P* < 0.0001, two-tailed Student’s *t* test, Fig. 4C). However, we observed no sex differences in DCP-induced pruritus and anti-pruritus effects of DMXAA between both sexes (Additional file 3: Fig. S3).

Our biochemical experiments found the significant decrease of spinal TBK1 and IRF3 phosphorylation (but not expression) on day 5 after DCP exposure (Fig. 4D–H). Intriguingly, DCP-induced reduction in TBK1 phosphorylation was inhibited by systemic DMXAA therapy (F (2, 15) = 117.7, *P* < 0.0001, one-way ANOVA, Fig. 4D and F). As parallel, the decreased IRF3 phosphorylation in ACD mice was elevated by DMXAA (F (2, 15) = 262.6, *P* < 0.0001, one-way ANOVA, Fig. 4D and H).

### Spinal decreases of STING expression in mice with morphine administration, dry skin and contact dermatitis are increased by DMXAA therapy

**Fig. 5:**
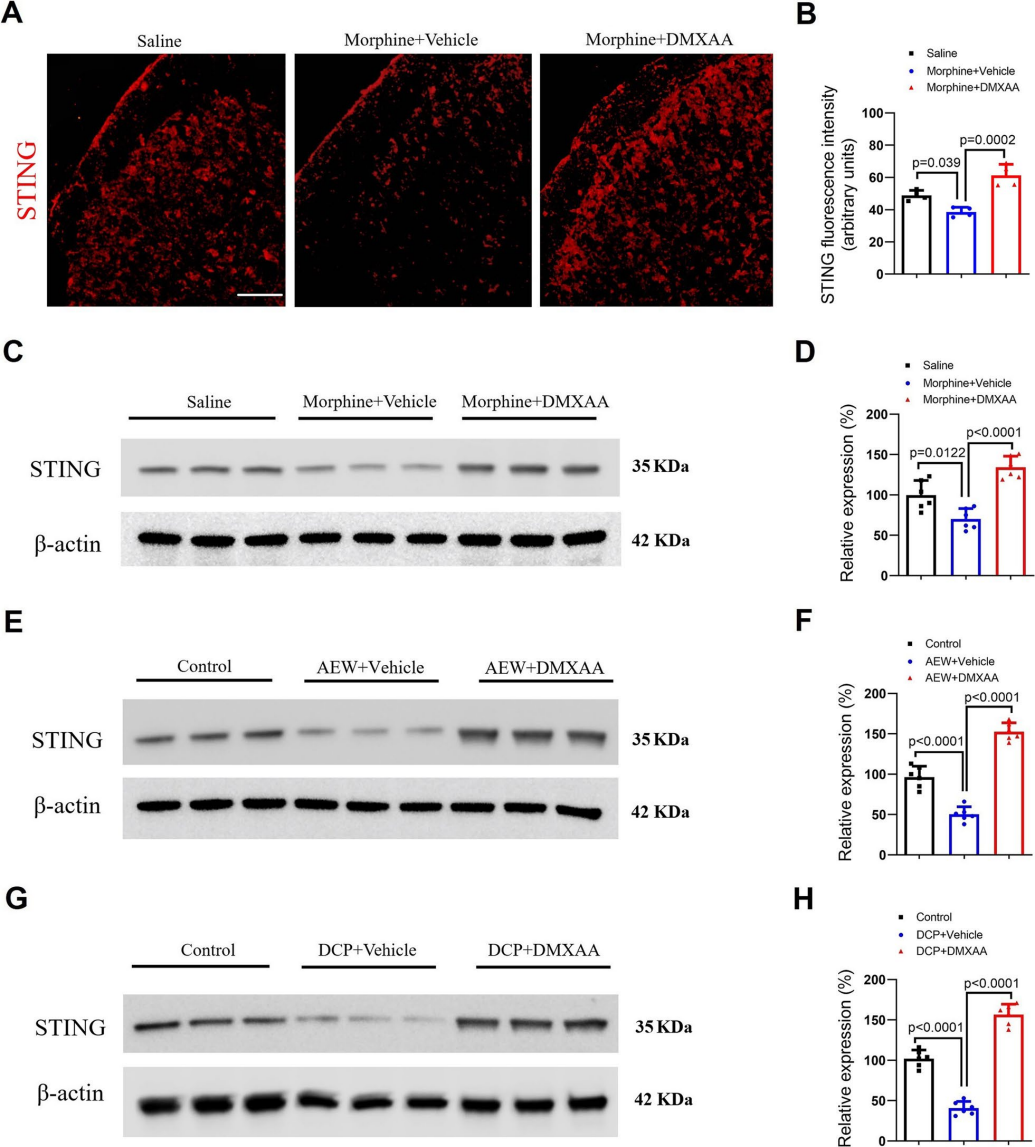
Spinal changes of STING expression after neuraxial morphine, dry skin and contact dermatitis. STING agonist DMXAA (i.p., 20 mg/kg) was administered 60 min prior to morphine (i.t., 0.3 nmol) exposure. **A** and **B** Immunostaining showed the fluorescence intensity of STING (red) in the spinal dorsal horn at 30 min after morphine injection. Scale bar, 100 μm. **C** and **D** Western blot showed that DMXAA treatment affects spinal STING expression at 30 min after morphine injection. **E** and **F** Western blot showed that two injections of DMXAA (i.p., 20 mg/kg) daily from day 1 to 2 after AEW exposure affect spinal STING expression on day 3 after AEW intervention. **G** and **H** Western blot showed that two injections of DMXAA (i.p., 20 mg/kg) daily from day 3 to 4 after DCP exposure affect spinal STING expression on day 5 after DCP intervention. n = 4–6 mice/group. Data are expressed as mean ± SD and analyzed by one-way ANOVA with Bonferroni post hoc test

We next examined the potential alternations of STING expression in the spinal dorsal horn in different mouse models of pruritus and systemic DMXAA intervention. Intriguingly, immunostaining detected that intrathecal morphine reduced spinal STING fluorescence intensity, which was prevented by DMXAA pre-treatment (F (2, 9) = 23.09, *P* = 0.0003, one-way ANOVA, Fig. 5A and B). More strikingly, Western blot also revealed that pre-administration of DMXAA significantly up-regulated the decreased expression of STING in the spinal dorsal horn of mice following intrathecal morphine injection (F (2, 15) = 26.76, *P* < 0.0001, one-way ANOVA, Fig. 5C and D), dry skin (F (2, 15) = 122.6, *P* < 0.0001, one-way ANOVA, Fig. 5E and F) and contact dermatitis (F (2, 15) = 177.7, *P* < 0.0001, one-way ANOVA, Fig. 5G and H). These further imply that spinal STING is important in opioid-induced acute itch and dermatitis-induced chronic itch.

### Pharmacological inhibition of TBK1-IFN-I signaling abolishes the anti-pruritus effects of DMXAA

**Fig. 6:**
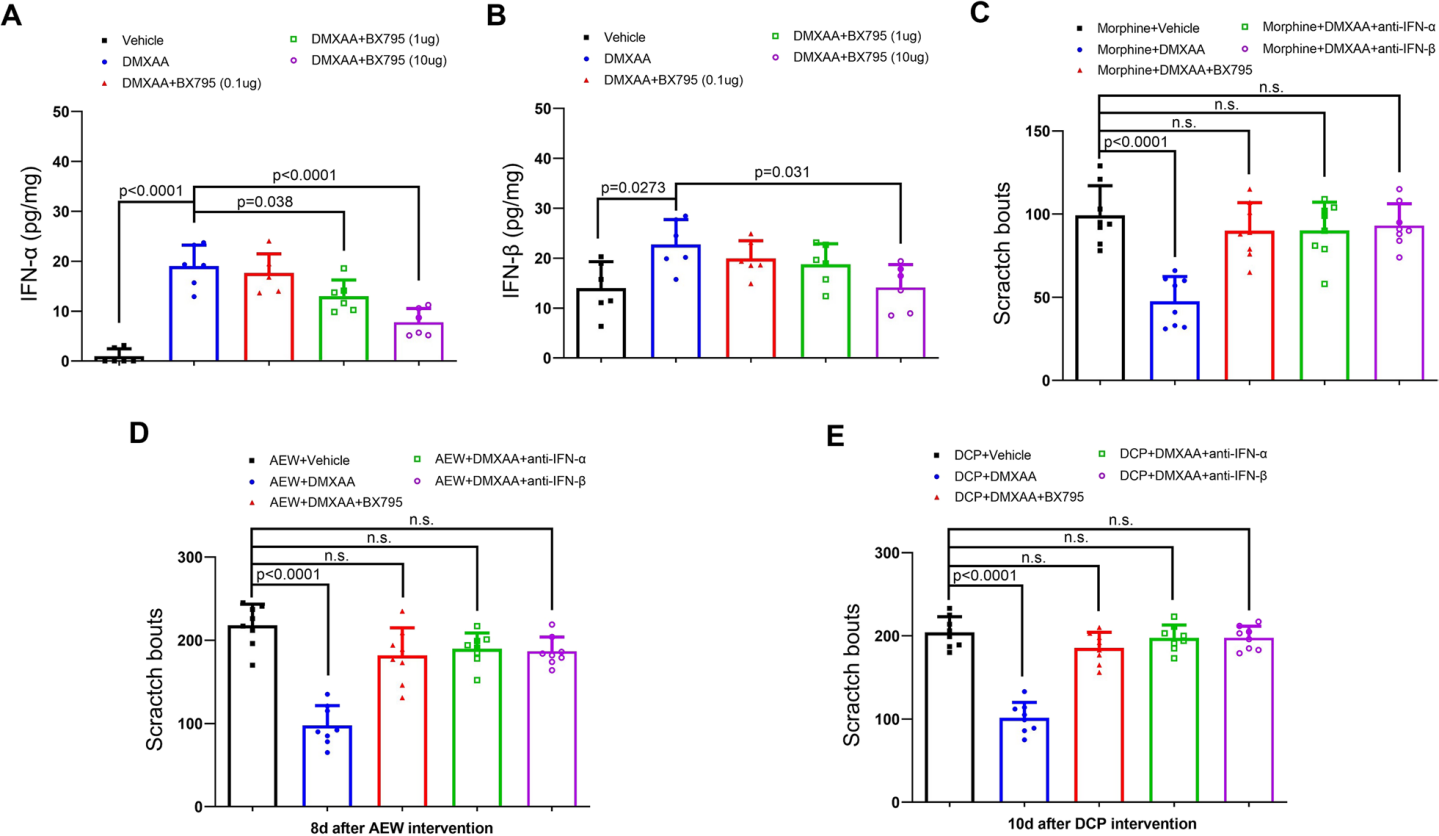
Pharmacological inhibition of IFN-I cascades impairs the anti-itch effects of STING agonism on opioid-induced acute itch and dermatitis-induce chronic itch in mice. **A** and **B** A selective TBK1 inhibitor BX795 (i.t., 0.1, 1 and 10 μg) was injected immediately after DMXAA (i.p., 20 mg/kg) treatment. ELISA assay showed the spinal concentrations of IFN-α and IFN-β at 1 h after DMXAA and BX795 treatment. n = 6 mice/group. **C-E** BX795 (i.t., 10 μg), anti-IFN-α (i.t., 300 ng) and anti-IFN-β (i.t., 300 ng) that were injected immediately after DMXAA (i.p., 20 mg/kg) treatment compensate the pruritus alleviation by DMXAA in three mouse models of morphine-induced itch (**C**), AEW-induced itch (**D**), and DCP-induced itch (**E**). n = 8 mice/group. Data are expressed as mean ± SD and analyzed by one-way ANOVA with Bonferroni post hoc test. n.s., not significant

Then, to investigate the potential role of canonical IFN-I cascade in STING activation and itch phenotype, a selective TBK1 inhibitor (BX795) and IFN-I neutralizing antibodies (anti-IFN-α and anti-IFN-β) were injected intrathecally and immediately after DMXAA treatment. We found that BX795 (1 and 10 μg but not 0.1 μg) effectively reduced the production of IFN-α by DMXAA treatment (F (4, 25) = 31.32, *P* < 0.0001, one-way ANOVA, Fig. 6A). Similarly, DMXAA-induced over-expression of IFN-β was restrained by BX795 (10 μg) co-administration (F (4, 25) = 4.171, *P* = 0.0101, one-way ANOVA, Fig. 6B). These data suggest that BX795 (10 μg) is sufficient to abolish spinal IFN-α and IFN-β production, thus we selected BX795 (10 μg) to block IFN-I cascades. As expected, behavioral experiments showed that BX795 (10 μg), anti-IFN-α (300 ng) and anti-IFN-β (300 ng) entirely compensated the therapeutic effects of DMXAA on morphine-induced itch (Fig. 6C), AEW-induced itch (Fig. 6D), and DCP-induced itch (Fig. 6E), manifesting that TBK1-dependent IFN-I response is a critical step for the alleviation of opioid-induced acute itch and dermatitis-induced chronic itch by STING agonism.

### ADU-S100 attenuates opioid-induced acute itch and dermatitis-induced chronic itch

**Fig. 7:**
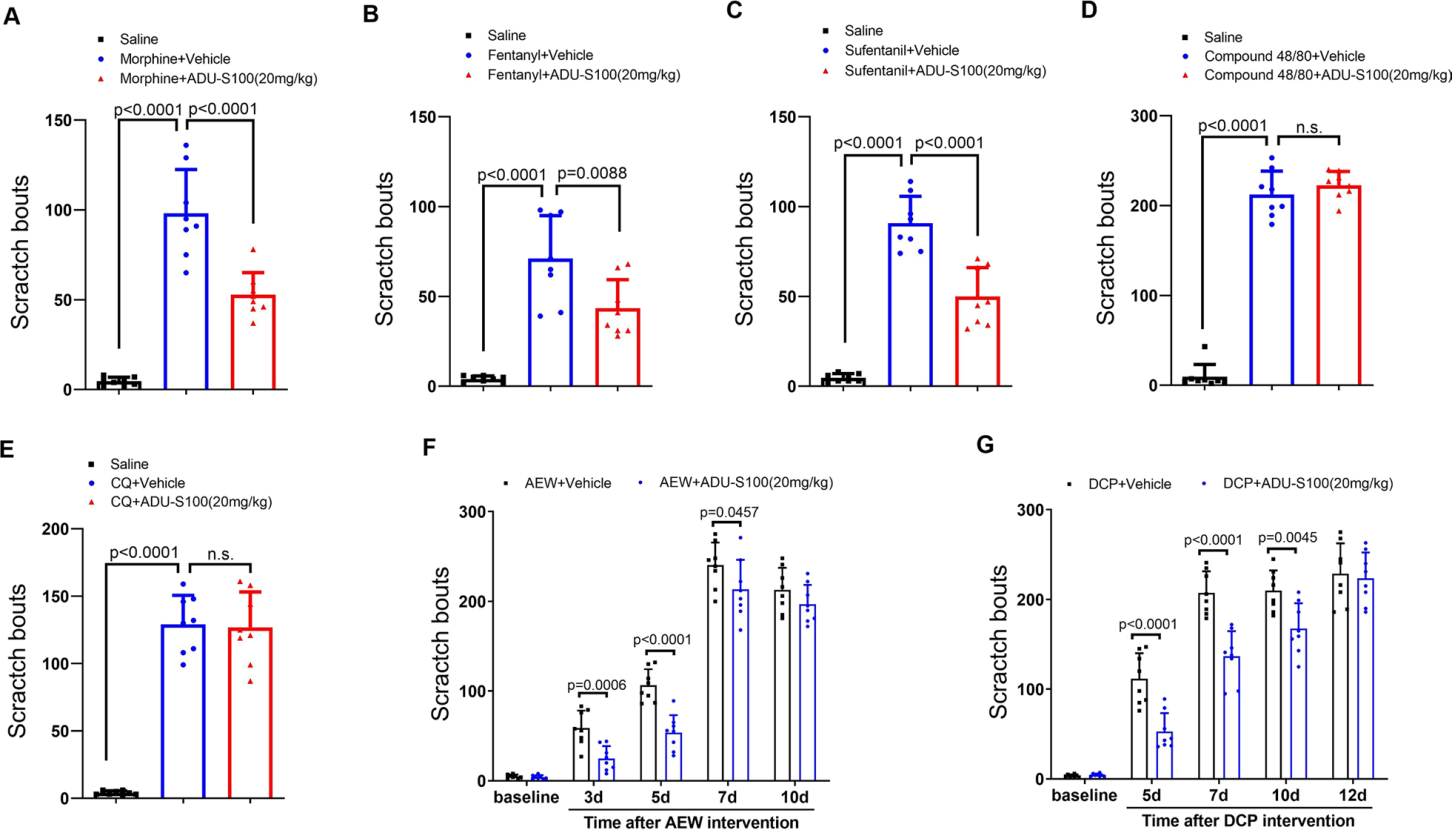
The cross-species STING agonist ADU-S100 reduces neuraxial opioids-induced acute itch and dermatitis-induce chronic itch in mice. **A**-**C** Intraperitoneal (i.p.) pre-administration of ADU-S100 attenuates i.t. morphine-, fentanyl-, and sufentanil-induced scratching behavior. **D** and **E** ADU-S100 does not affect i.d. compound 48/80- and CQ-induced scratching behavior. **F** pre-treatment with ADU-S100 reduces AEW-induced scratching behavior. **G** pre-treatment with ADU-S100 reduces DCP-induced scratching behavior. Data are expressed as mean ± SD and analyzed by one-way ANOVA with Bonferroni post hoc test (**A**–**E**), and two-way ANOVA with Bonferroni post hoc test (**F**, **G**). n = 8 mice/group. n.s., not significant

Recently, the cross-species STING agonist ADU-S100 has showed encouraging activity and safety profile in several ongoing clinical trials [29]. Given the limited translational significance of DMXAA in clinical patients because of its specificity to murine STING [22], we also explored whether ADU-S100 could exhibit similar therapeutic properties in different models of acute and chronic pruritus. First, i.t. morphine-induced itch behavior was drastically diminished by ADU-S100 (i.p., 20 mg/kg) pre-administration (F (2, 21) = 69.15, *P* < 0.0001, one-way ANOVA, Fig. 7A). Similarly, systemic ADU-S100 therapy prevented neuraxial fentanyl- and sufentanil-induced pruritus (F (2, 21) = 32.62, *P* < 0.0001, and F (2, 21) = 90.43, *P* < 0.0001, respectively, one-way ANOVA, Fig. 7B and C). However, pruritogens-induced acute pruritus was not altered by pre-treatment with ADU-S100 (Fig. 7D and E). Notably, AEW-induced chronic itch in dry skin model was successfully restrained by i.p. delivery of ADU-S100 (F (1, 70) = 33.46, *P* < 0.0001, two-way ANOVA, Fig. 7F). Systemic ADU-S100 therapy was also sufficient to reduce DCP-induced chronic itch in ACD model (F (1, 70) = 41.91, *P* < 0.0001, two-way ANOVA, Fig. 7G). Collectively, these behavioral data further illustrated that STING agonism controls opioid-induced acute itch and dermatitis-induced chronic itch.

### Recombinant IFN-I suppresses intrathecal opioid-induced acute itch and dermatitis-induced chronic itch

**Fig. 8:**
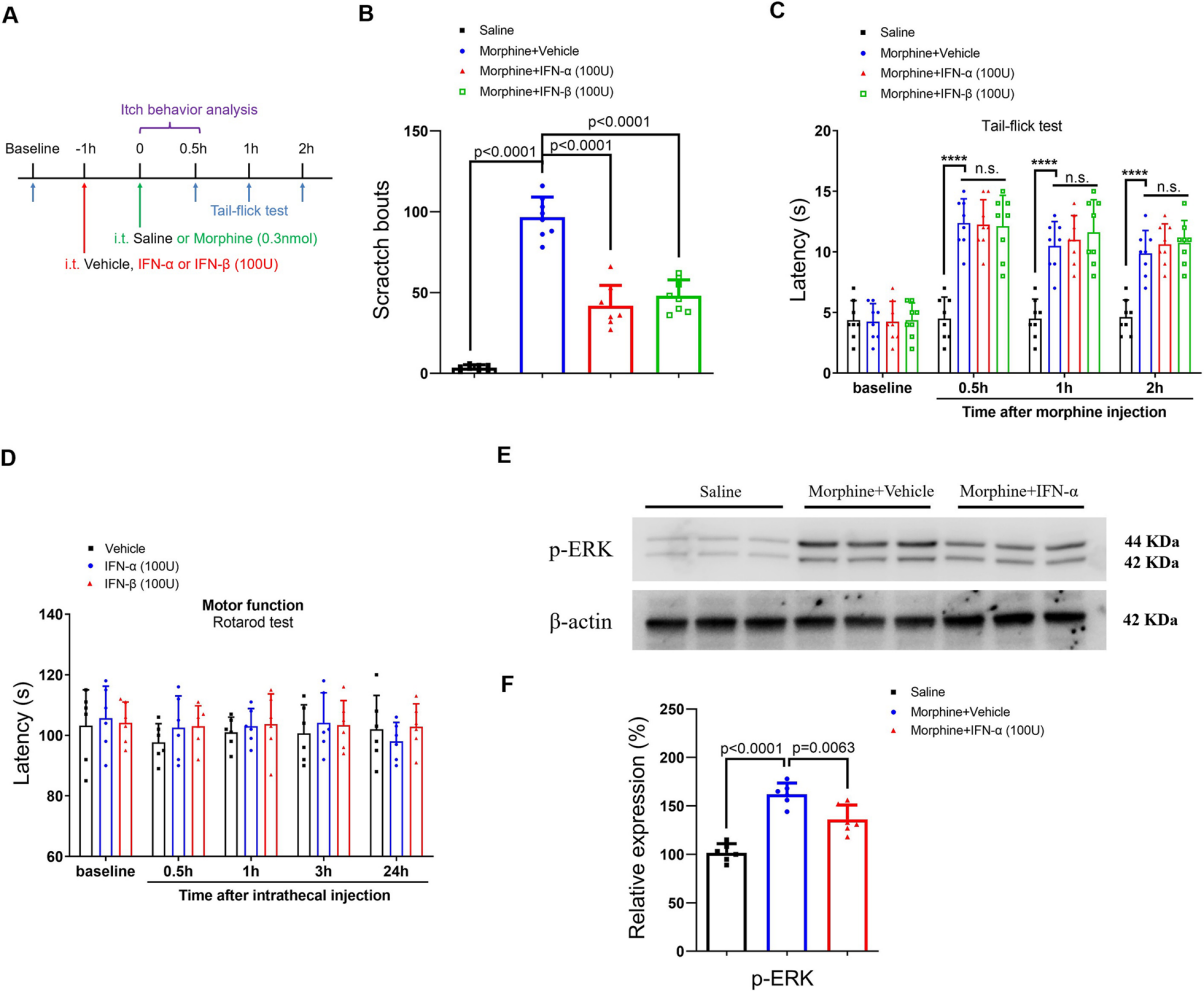
IFN-I activation reduces neuraxial opioid-induced acute itch and spinal phosphorylation of ERK in mice. **A** Experimental design for recombinant IFN-α and IFN-β treatment in intrathecal (i.t.) opioid-induced itch. **B** pre-administration of recombinant IFN-α and IFN-β attenuates i.t. morphine-induced scratching behaviors. n = 8 mice/group. **C** Morphine antinociception, assessed by Tail-flick test, is not impaired by recombinant IFN-α and IFN-β therapy. n = 8 mice/group. **D** Locomotor function, assessed by Rotarod test, is unaffected by recombinant IFN-α and IFN-β therapy. n = 6 mice/group. **E** and **F** Western blot showed that recombinant IFN-α treatment inhibits spinal phosphorylation of ERK at 30 min after morphine injection. n = 6 mice/group. Data are expressed as mean ± SD and analyzed by one-way ANOVA with Bonferroni post hoc test (**B** and **F**), and two-way ANOVA with Bonferroni post hoc test (**C** and **D**). Compared with group Saline, ^****^P < 0.0001. n.s., not significant

**Fig. 9:**
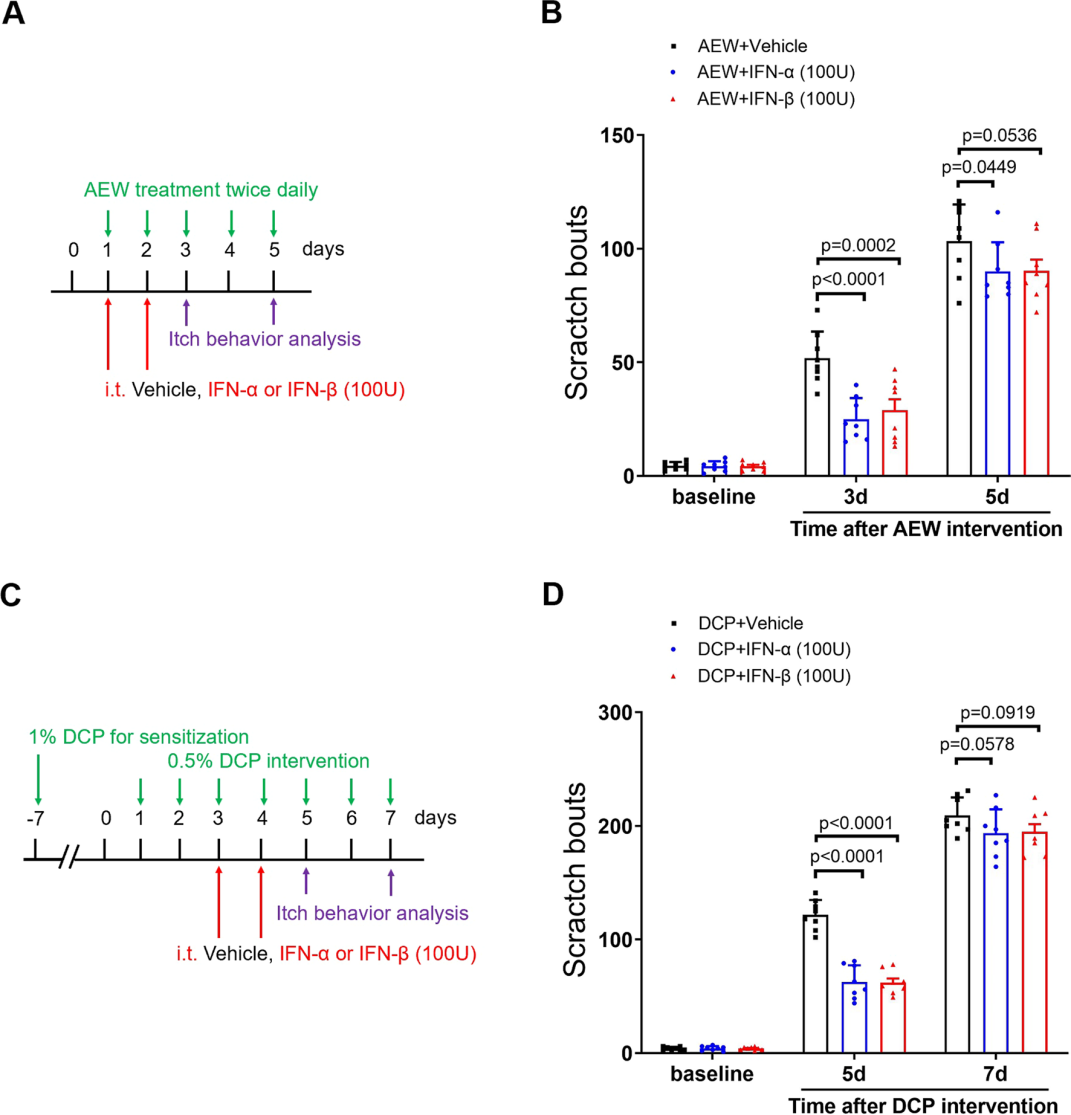
IFN-I activation reduces dry skin- and dermatitis-induce chronic itch in mice. **A** Experimental design for recombinant IFN-α and IFN-β therapy in AEW-induced chronic pruritus. **B** Pre-treatment with recombinant IFN-α and IFN-β reduces AEW-induced scratch bouts. **C** Experimental design for recombinant IFN-α and IFN-β therapy in DCP-induced chronic pruritus. **D** Pre-treatment with recombinant IFN-α and IFN-β reduces DCP-induced scratch bouts. n = 8 mice/group. Data are expressed as mean ± SD and analyzed by two-way ANOVA with Bonferroni post hoc test

Next, we sought to test the direct effect of IFN-I on central pruriceptive sensitization in itch conditions. To tackle this, recombinant IFN-α (i.t., 100 U) and IFN-β (i.t., 100 U) were administered. Herein, we found that recombinant IFN-α and IFN-β therapy effectively reduced i.t. morphine-induced acute itch (F (3, 28) = 112.9, *P* < 0.0001, one-way ANOVA, Fig. 8A and B) without compromising morphine analgesic action (Fig. 8C) and locomotor function (Fig. 8D). The phosphorylation of extracellular signal-regulated kinase (ERK) is a cardinal manifestation of central pruriceptive sensitization in the spinal transmission of itch sensation [30]. Consistent with a previous report [31], our biochemical results exhibited the rapid phosphorylation of spinal ERK at 30 min following neuraxial morphine administration (Fig. 8E and F). Surprisingly, pretreatment with recombinant IFN-α down-regulated the increase of phosphorylated ERK due to acute exposure to morphine (F (2, 15) = 37.76, *P* < 0.0001, one-way ANOVA, Fig. 8E and F). Also, recombinant IFN-α and IFN-β attenuated AEW-induced chronic itch (F (2, 63) = 11.48, *P* < 0.0001, two-way ANOVA, Fig. 9A and B) and DCP-induced chronic itch (F (2, 63) = 28.45, *P* < 0.0001, two-way ANOVA, Fig. 9C and D). Thus, these results suggest that IFN-I activation protects against opioid-induced itch and chronic itch through spinal inhibition of ERK phosphorylation.

### DMXAA and recombinant IFN-I attenuate compound 48/80- and dry skin-induced alloknesis

**Fig. 10:**
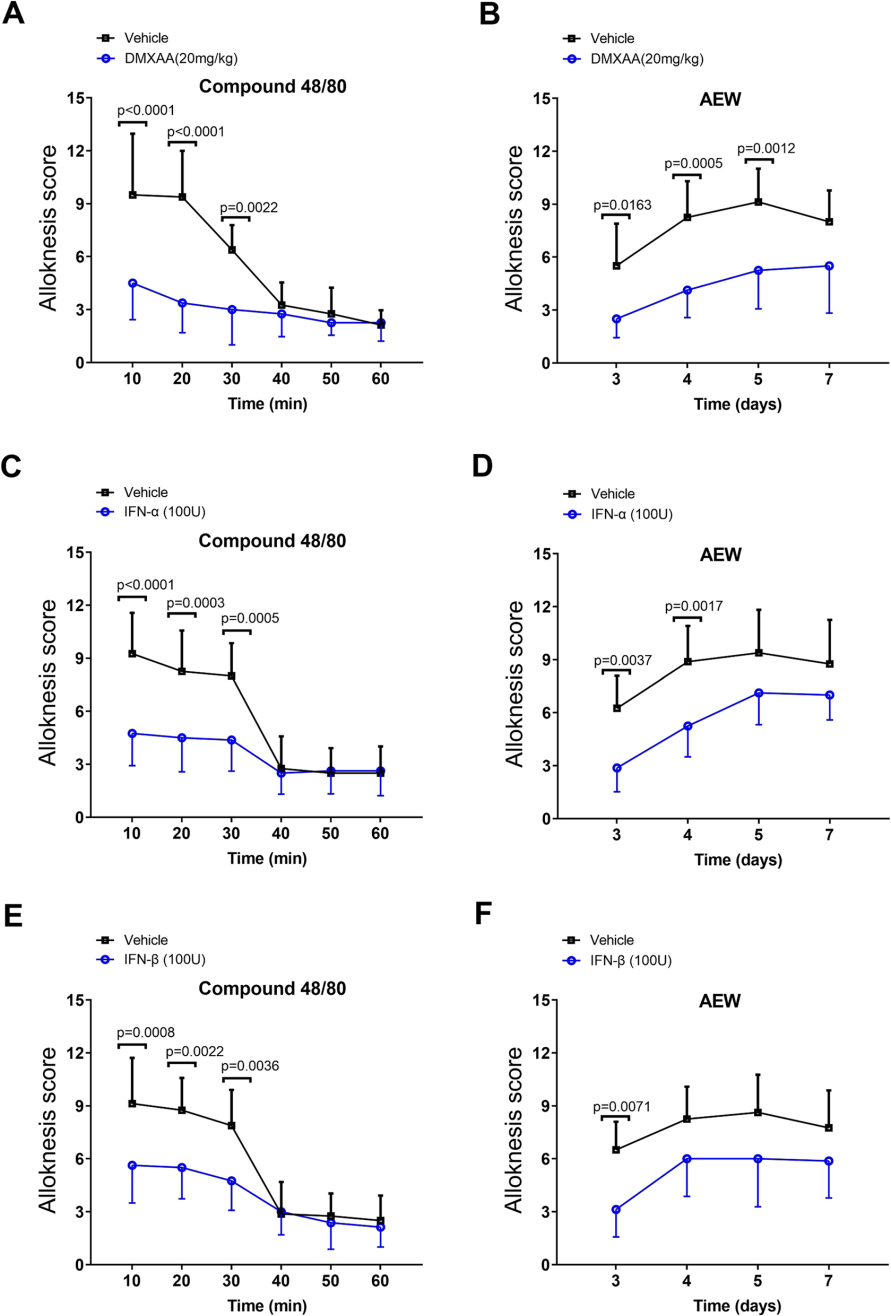
The activation of STING-dependent IFN-I cascades reduces acute and chronic alloknesis in mice. **A** DMXAA (i.p., 20 mg/kg) is injected at 60 min prior to compound 48/80 exposure. Alloknesis, induced 30 min after compound 48/80 treatment, is reduced by DMXAA pre-treatment. **B** Two injections of DMXAA (i.p., 20 mg/kg) are given daily from day 1 to 2 after AEW treatment. Alloknesis, induced after AEW-induced dry skin, is reduced by DMXAA pre-treatment. **C** and **E** Recombinant IFN-α and IFN-β (i.t., 100 U) are injected at 60 min prior to compound 48/80 exposure. Alloknesis, induced 30 min after compound 48/80 treatment, is reduced by recombinant IFN-α and IFN-β. **D** and **F** Two injections of recombinant IFN-α and IFN-β (i.t., 100 U) are given daily from day 1 to 2 after AEW treatment. Alloknesis, induced after AEW-induced dry skin, is reduced by recombinant IFN-α and IFN-β. Data are expressed as mean ± SD and analyzed by two-way ANOVA with Bonferroni post hoc test. n = 8 mice/group. n.s., not significant

Touch-elicited pruritus, referred to as alloknesis or mechanical itch, is mediated by central sensitization [32]. We scored alloknesis by counting scratch bouts following application of 0.7 mN von Frey stimuli 30 min after intradermal delivery of compound 48/80[33]. Systemic DMXAA therapy at 60 min prior to compound 48/80 exposure exhibited a considerable decrease in alloknesis score during a 60-min period (F (1, 84) = 46.85, *P* < 0.0001, two-way ANOVA, Fig. 10A). Meanwhile, alloknesis was evoked in mice with AEW intervention, observed from day 3 and peaked on day 5 (Fig. 10B). As expected, dry skin-evoked development of alloknesis was restricted by two injections of DMXAA daily from day 1 to 2 after AEW treatment (F (1, 56) = 45.41, *P* < 0.0001, two-way ANOVA, Fig. 10B). Moreover, recombinant IFN-α (i.t., 100 U) was able to diminish compound 48/80-induced acute alloknesis (F (1, 84) = 30.42, *P* < 0.0001, two-way ANOVA, Fig. 10C) and AEW-evoked chronic alloknesis (F (1, 56) = 32.42, *P* < 0.0001, two-way ANOVA, Fig. 10D). In addition, recombinant IFN-β (i.t., 100 U) showed similar therapeutic effects on alloknesis in our mouse models (Fig. 10E and F). These results suggest that activation of STING-dependent IFN-I cascades exerts an inhibitory effect on alloknesis after acute pruritus and during chronic pruritus.

### DMXAA prevents dermatitis-induced the alternations of brain functional connectivity between ROIs

**Fig. 11:**
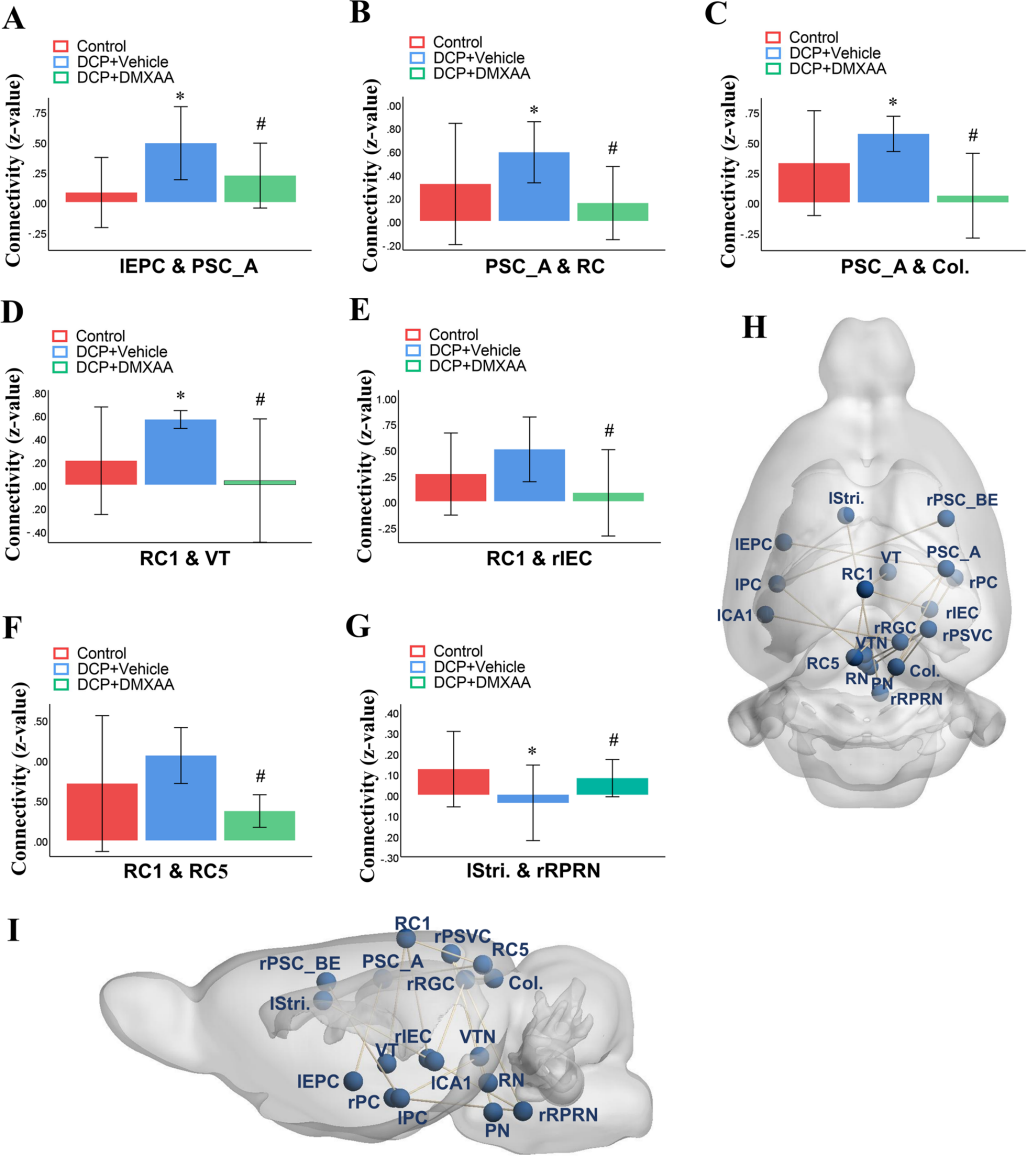
The murine STING agonist DMXAA prevents the changes of brain functional connectivity in dermatitis-induced chronic pruritus in mice. DMXAA (i.p., 20 mg/kg) was injected daily from days 3 to 4 after DCP treatment in contact dermatitis model. **A**–**G** Regions of interest (ROI)-ROI functional connectivity differences are examined between different groups on day 5 following DCP. The vertical axes represent the z-value of the ROI-ROI functional connectivity. **H** and **I** 3D representation of whole-brain ROI-ROI functional connectivity. n = 3 mice/group. Data are expressed as mean ± SD and analyzed by one-way ANOVA with Bonferroni post hoc test. Compared with group Control, ^*^P < 0.05. Compared with group DCP + Vehicle, ^#^*P* < 0.05. lCA1, left cornu ammonis 1; Col, colliculus; lEPC, left endo/piriform cortex; rIEC, right intermedial entorhinal cortex; rPC, right piriform cortex; lPC, Left piriform cortex; PN, pontine nuclei; PSC_A, primary somatosensory cortex (auditory); rPSC_BE, right primary somatosensory cortex (BE); rPSVC, right primary and secondary visual cortex; RC1, retrosplenial cortex 1; RC5, retrosplenial cortex 5; RN, raphe nuclei; rRPRN, right raphe pontine reticular nuclei; lStri, left striatum; VT, ventral thalamus; VTN, ventral tegmental nuclei

fMRI-based neuroimaging is a most informative and noninvasive approach to elucidate brain function in physiological and pathological conditions in humans [34]. There is accumulating interest in back-translating this approach to basic research in animals to provide mechanistic insights into the development of neurological disorders on whole-brain activity and functional connectivity in vivo [35]. fMRI is also important in evaluation of therapeutic effects after drugs administration and ultimately enhance the success rate of drugs discovery in central nervous system research [36]. Thus, we employed resting-state fMRI to assess brain activity and responses to STING agonist in dermatitis-induced chronic itch model (Fig. 11). Intriguingly, we found the obvious increase of cerebral functional connectivity between primary somatosensory cortex and left piriform cortex, retrosplenial cortex 1, and colliculus on 5 days following DCP exposure (Fig. 11A–C). Similarly, we detected the increase of cerebral functional connectivity between retrosplenial cortex 1 and ventral thalamus, right intermedial entorhinal cortex, and retrosplenial cortex 5 (Fig. 11D-F). Also, we revealed the decrease of cerebral FC between left striatum and right paramedian pontine reticular nuclei (Fig. 11G). More importantly, these functional network changes were drastically prevented by systemic DMXAA therapy (i.p., 20 mg/kg) daily from days 3 to 4 (in the early phase) after DCP treatment (Fig. 11).

## Discussion

The central findings of the present investigation are: First, STING agonism alleviates neuraxial opioid-induced itching-like behavior, and increases spinal TBK1 and IRF3 phosphorylation. Second, systemic STING agonism inhibits the initiation and maintenance of dry skin-induced chronic pruritus and contact dermatitis-induced chronic pruritus. Spinal down-regulations of TBK1 and IRF3 phosphorylation in these two chronic itch models are elevated by STING activation. Third, pharmacological suppression of TBK1-IFN-I cascades is sufficient to abolish the anti-itch effects of STING agonism. Fourth, IFN-I activation is effective in treating opioid-induced acute itch, dry skin-induced chronic itch, as well as contact dermatitis-induced chronic pruritus. Exogenous IFN-α is capable of inhibiting spinal ERK phosphorylation following acute exposure to morphine. Fifth, STING agonism and IFN-I activation impair pruritogen- and dry skin-evoked alloknesis. Sixth, cerebral functional network changes between major itch pathways in contact dermatitis model are diminished by STING activation. These findings therefore recapitulate a unique and unrecognized advantage of STING agonism in itch treatment by the activation of TBK1-IRF3-dependent IFN-I response, suggesting that STING agonists can be used as a promising therapeutic approach for the management of pruritus with different etiologies.

Opioids are frequently added to neuraxial analgesia to minimize the use of local anesthetics, which diminishes hemodynamic fluctuation and hastens motor block recovery but at a cost of an elevated risk of pruritus [7]. According to statistics, the incidence of pruritus in non-obstetric patients receiving neuraxial morphine is 15%-70% and neuraxial fentanyl or sufentanil is 53%-79%, whereas the incidence increases to 60%-100% of obstetric patients after intrathecal opioids treatment [6, 7]. Despite decades of clinical investigation and medical advancement, current approaches for opioid-induced pruritus relief are still limited. 5-hydroxytryptamine3 receptor antagonists are only partially effective and opioid antagonists often compromise spinal opioids analgesia [6, 37]. Thus, alternative agents for pruritus control are urgently required. Recently, STING agonists show efficacy in treating tumor progression, ischemic stroke, depression, and pathological pain [17, 18, 21, 38]. Herein, we provide the first evidence that STING agonists (DMXAA and ADU-S100) drastically inhibits intrathecal opioid (morphine, fentanyl and sufentanil)-induced pruritus and these benefits do not come at the price of impairing optimal opioids antinociception and locomotor function.

Compound 48/80-induced acute pruritus is primarily associated with mast-cell degranulation and subsequent histamine release, which could be eliminated by antagonism of histamine receptor H1 and H4 [39]. CQ-evoked acute pruritus is mainly generated by MrgprA3 (Mas-related G-protein coupled receptor member A3), for which anti-histamine drugs are ineffective [27]. Chronic pruritus often originates from inflammatory skin diseases, such as dry skin, contact dermatitis and atopic dermatitis [9]. Chronic pruritus (but not pruritogen-induced acute pruritus) is demonstrated to be attributed to central pruriceptive sensitization, as mechanoreceptors activation excites sensitized pruritus-signaling neurons in the spinal dorsal horn [32, 33, 40]. Our present study, for the first time, reveals that STING agonism is effective in alleviating chronic itch caused by dry skin and contact dermatitis, whereas it is ineffective in reducing pruritogens (compound 48/80 and CQ)-induced acute itch. Moreover, we observe the primary expression of STING protein in the spinal dorsal horn neurons (but not astrocyte and microglia). Mechanistically, these strongly imply a critical role of STING activation in inhibiting central pruriceptive sensitization, neuronal excitability and neural plasticity. Supporting these data, recent reports recapitulate that STING agonists protect against pathological pain via direct modulation of neuronal activity [21, 22]. Nevertheless, the specific molecular mechanism underlying the contribution of STING agonism to opioid-induced itch and chronic itch remains largely undefined.

Activated STING is required for the production of IFN-I via STING-TBK1-IRF3 cascades in pathophysiological conditions [15, 16]. Dysregulation of STING/IFN-I signaling causes neurotoxicity and neuroinflammation, which is a leading determinant in the neurobiology of diseases [41]. In contrast, STING-mediated IFN-I response is revealed to be protective against depression-like behaviors after chronic restraint stress and experimental autoimmune encephalomyelitis in a mouse model of multiple sclerosis [17, 19]. Consistently, other studies also confirmed that the therapeutic properties of STING agonists on bone cancer pain, fracture-associated pain, and neuropathic pain are dependent on IFN-I signaling [21, 22]. Herein, we provided several lines of evidence to support that IFN-I signaling is one of the most essential downstream effectors of STING activation for the treatment of opioid-induced itch and chronic itch. First, spinal expression of STING and spinal phosphorylation of TBK1 and IRF3 are decreased in mice with morphine injection, dry skin and contact dermatitis. Second, STING agonism increases spinal STING-dependent phosphorylation of TBK1 and IRF3 in morphine-induced acute itch and dermatitis-induced chronic itch. Third, systemic STING agonism induces the production of spinal IFN-I. Moreover, intrathecal treatment with exogenous IFN-I recapitulated all anti-pruriceptive effects of STING agonists. Fourth, spinal TBK1 inhibition reduces STING-dependent IFN-I response. Fifth, STING activation-mediated anti-itch is all compromised and abolished by TBK1 inhibitor and IFN-I neutralizing antibodies. Mechanistically, our study has provided a novel mechanistic insight into neuromodulation of opioid-induced itch and chronic itch. While our findings elucidate that STING agonism may be a beneficial approach for activating TBK1-dependent IFN-I response for controlling pathologic itch states, further investigations are warranted to establish specific mechanistic links between these molecular changes, behavioral phenotypes and neural circuitry dysfunction in pruritus.

Previous views proposed that morphine activates the mu opioid receptor isoform 1D (MOR1D), further generates the heterodimerization of MOR1D and the gastrin-releasing peptide receptor (GRPR), and subsequently enhances the excitability of GRPR-expressing pruriceptive neurons, contributing to itch sensation [42]. A recent literature by Wang and colleagues demonstrated that intrathecal administration of morphine acts upon the mu opioid receptor (MOR) from spinal GABAergic inhibitory interneurons to elicit itch through disinhibiting the activities of excitatory pruriceptive neurons expressing GRPR [11]. Another study revealed that binding morphine to MOR on dynorphin-expressing spinal inhibitory neurons is both necessary and sufficient for the development of itch-like behaviors [43]. Collectively, neuraxial opioid inhibits a subset of spinal itch-inhibiting neurons, resulting in the subsequent disinhibition of excitatory pruriceptive neurons, thereby evoking pruritus. Our current findings imply that intrathecal morphine injection, dry skin and contact dermatitis might inhibit spinal STING expression to reduce the phosphorylation of TBK1 and IRF3, causing acute and chronic itch. However, whether MOR is implicated in STING inhibition in the pathogenesis of spinal itch processing remains elusive. The main limitation of this study is inability to elucidate whether spinal STING is expressed on MOR-expressing neurons and test the functional interaction between STING and MOR. Another question arising from our work is how IFN-I activation associated with STING agonism attenuates opioid-induced itch. One possibility is that STING-dependent IFN-I response facilitates the activity of spinal itch-inhibiting neurons to inhibit the excitability of itch-encoding neurons, leading to itch-relief. Sure, one possible weakness is that we failed to investigate whether spinal STING is expressed on (GABAergic and dynorphin-expressing) inhibitory neurons and test the role of STING activation in inhibitory neurons. In addition, in this current study, Immunostaining revealed the co-localization of STING protein with dorsal horn neurons, but there is also extensive positive staining outside of the neurons. This immunopositivity imply that spinal STING protein may be also expressed on other cellular population, such as fibroblasts, endothelial cells, and immune cells. However, whether STING protein in these types of cells regulates itch remains unexplored, we should take this into account in future study.

Light touch at the normal skin surrounding an itchy site may induce alloknesis, which is commonly detectable in patients with chronic itch syndromes [10, 32, 33]. Previous studies have manifested the requirement of central sensitization in the spinal dorsal horn neurons for the pathogenesis of alloknesis [32, 40]. In our present study, a striking behavioral finding is that STING agonism and IFN-I activation abrogate compound 48/80-induced acute alloknesis and dry skin-evoked chronic alloknesis, suggesting the potential interaction between STING-dependent IFN-I response and neuromodulation (central pruriceptive sensitization) in spinal transmission of itch. Phosphorylation of ERK is gradually recognized as one of the most crucial contributors in the modifications of sensitized itch-signaling neurons, which underlies neural plasticity and central sensitization of refractory pruritus [30, 31]. To the best of our knowledge, this is the first report in which recombinant IFN-α reduces spinal phosphorylation of ERK in mice with neuraxial opioid-induced acute itch. An intriguing suggestion that arises from this present study is that the suppression of phosphorylated ERK-mediated central sensitization is implicated in the anti-pruritus mechanism of STING agonists for the management of pathological itch. Given that blocking GRPR by pharmacological or genetic approaches has been identified to alleviate prolonged pruritus via ablating spinal phosphorylation of ERK in mice with squaric acid dibutylester-induced contact dermatitis [30], it will be of great interest to determine whether IFN-I restricts ERK activation via the modulation of GRPR on spinal itch-responsive neurons in our itch models. Moreover, the identification of ERK activation-mediated transcriptional processing would become a rational step in clarifying how persistent phosphorylation of ERK encodes sustained itch behaviors. In addition, given that STING in nociceptive primary sensory neurons controls pain states [21], further experiments are also required to test whether STING in pruriceptive primary sensory neurons may play an important role in antipruritic effects.

In conclusion, the current findings uncover an unconventional pharmacological role of STING agonists in the alleviation of opioid-induced itch and dermatitis-induced chronic itch by regulating spinal TBK1-IRF3-dependent IFN-I response, ERK phosphorylation and subsequent cerebral functional connectivity in mice. Consequently, STING agonism and IFN-I activation may emerge as promising therapeutic avenues for pruritus relief in patients with opioid treatment and dermatitis.

## Supplementary Information

**Additional file 1: Figure S1**. The murine STING agonist DMXAA reduces intrathecal opioid-induced pruritus in both male and female mice. **A** Intraperitoneal pre-administration of DMXAA attenuates i.t. morphine-induced scratching behaviors in a dose-dependent manner in both sexes. Compared with group Saline, ****P < 0.0001. Compared with group Morphine + Vehicle, ^##^P < 0.01, ^####^P < 0.0001. **B** and **C** Fentanyl- and sufentanil-induced scratching behaviors in male and female mice are equally alleviated by DMXAA treatment. Compared with group Saline, ****P < 0.0001. Compared with group Fentanyl/Sufentanil, ^####^P < 0.0001. n = 6 mice/group. Data are expressed as mean ± SD and analyzed by two-way ANOVA with Bonferroni post hoc test. n.s., not significant.

**Additional file 2: Figure S2**. The murine STING agonist DMXAA reduces dry skin-induced pruritus in both male and female mice. Mice received two injections of DMXAA daily from day 1 to 2 after AEW intervention. AEW-induced chronic scratching behaviors in male and female mice are equally alleviated by DMXAA treatment. Compared with baseline, ****P < 0.0001. Compared with group AEW + Vehicle, ^####^P < 0.0001. n = 6 mice/group. Data are expressed as mean ± SD and analyzed by two-way ANOVA with Bonferroni post hoc test. n.s., not significant.

**Additional file 3: Figure S3**. The murine STING agonist DMXAA reduces contact dermatitis-induced pruritus in both male and female mice. Mice received two injections of DMXAA daily from day 3 to 4 after DCP intervention. DCP-induced chronic scratching behaviors in male and female mice are equally alleviated by DMXAA treatment. Compared with baseline, ****P < 0.0001. Compared with group DCP + Vehicle, ^####^P < 0.0001. n = 6 mice/group. Data are expressed as mean ± SD and analyzed by two-way ANOVA with Bonferroni post hoc test. n.s., not significant.

## Data Availability

All data relevant to the study are included in the article for figures and Additional figures. Data are available from the corresponding author upon reasonable request.
