# Supplementary figures and images for: STING controls opioid-induced itch and chronic itch via spinal tank-binding kinase 1-dependent type I interferon response in mice
Source: J Neuroinflammation. 2023 Apr 30;20:101. doi: 10.1186/s12974-023-02783-0 (PMC10150483; doi:10.1186/s12974-023-02783-0)

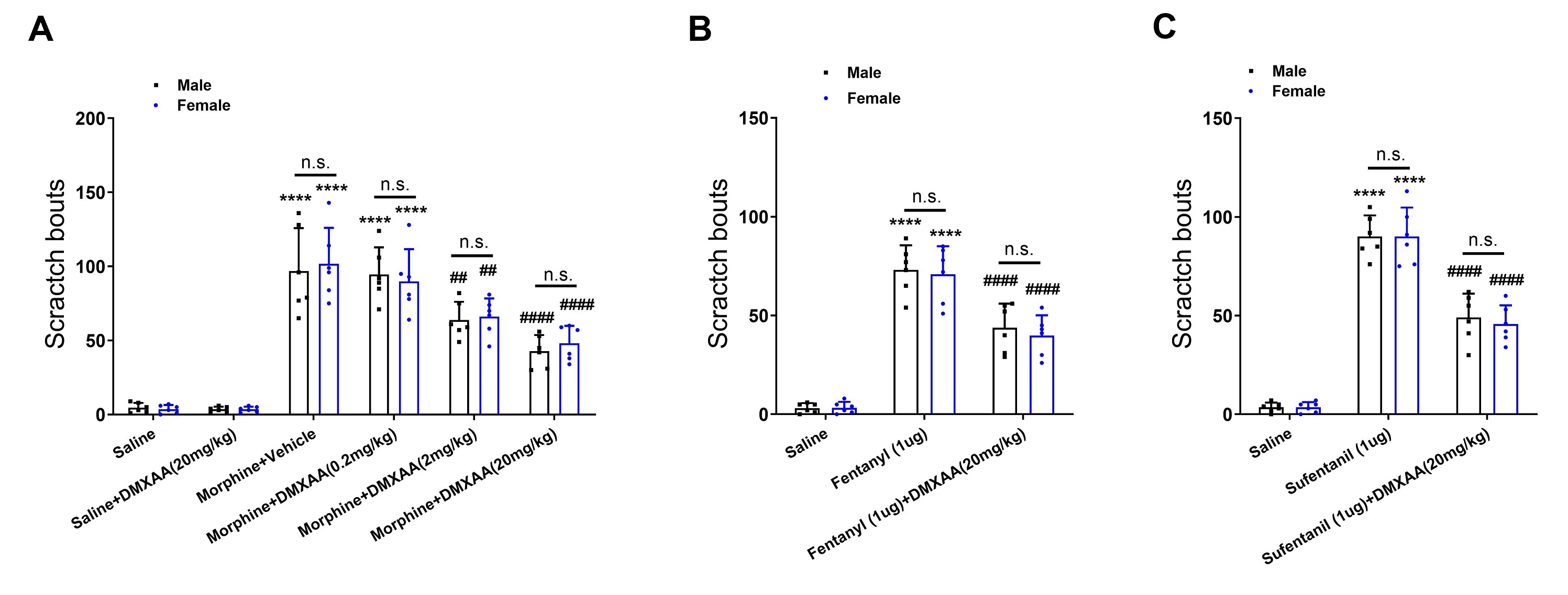

Supplement: Supplementary file 1 — Additional file 1: Figure S1. The murine STING agonist DMXAA reduces intrathecal opioid-induced pruritus in both male and female mice. A Intraperitoneal pre-administration of DMXAA attenuates i.t. morphine-induced scratching behaviors in a dose-dependent manner in both sexes. Compared with group Saline, ****P < 0.0001. Compared with group Morphine + Vehicle, ##P < 0.01, ####P < 0.0001. B and C Fentanyl- and sufentanil-induced scratching behaviors in male and female mice are equally alleviated by DMXAA treatment. Compared with group Saline, ****P < 0.0001. Compared with group Fentanyl/Sufentanil, ####P < 0.0001. n = 6 mice/group. Data are expressed as mean ± SD and analyzed by two-way ANOVA with Bonferroni post hoc test. n.s., not significant. [file 12974_2023_2783_MOESM1_ESM.jpg]

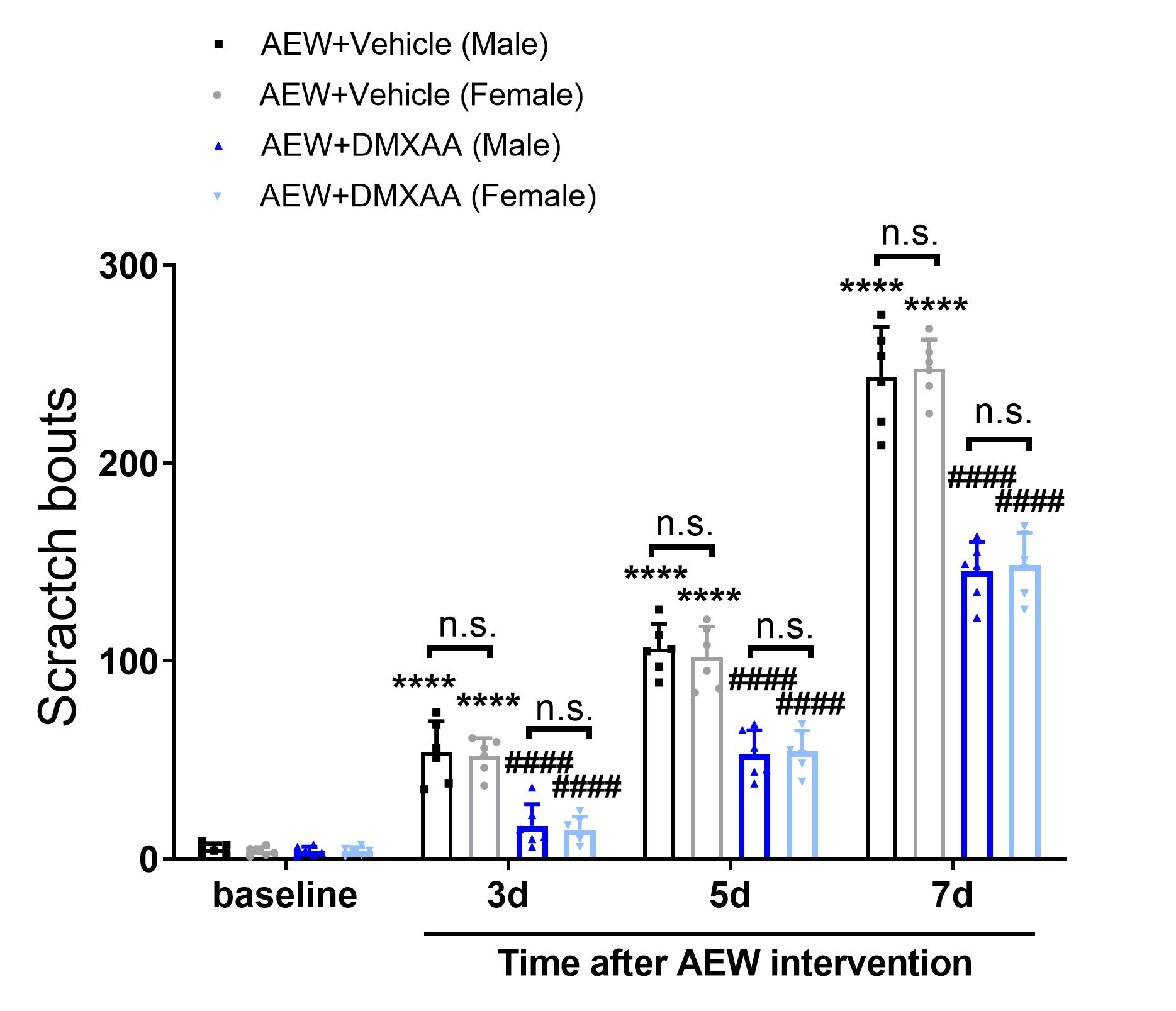

Supplement: Supplementary file 2 — Additional file 2: Figure S2. The murine STING agonist DMXAA reduces dry skin-induced pruritus in both male and female mice. Mice received two injections of DMXAA daily from day 1 to 2 after AEW intervention. AEW-induced chronic scratching behaviors in male and female mice are equally alleviated by DMXAA treatment. Compared with baseline, ****P < 0.0001. Compared with group AEW + Vehicle, ####P < 0.0001. n = 6 mice/group. Data are expressed as mean ± SD and analyzed by two-way ANOVA with Bonferroni post hoc test. n.s., not significant. [file 12974_2023_2783_MOESM2_ESM.jpg]

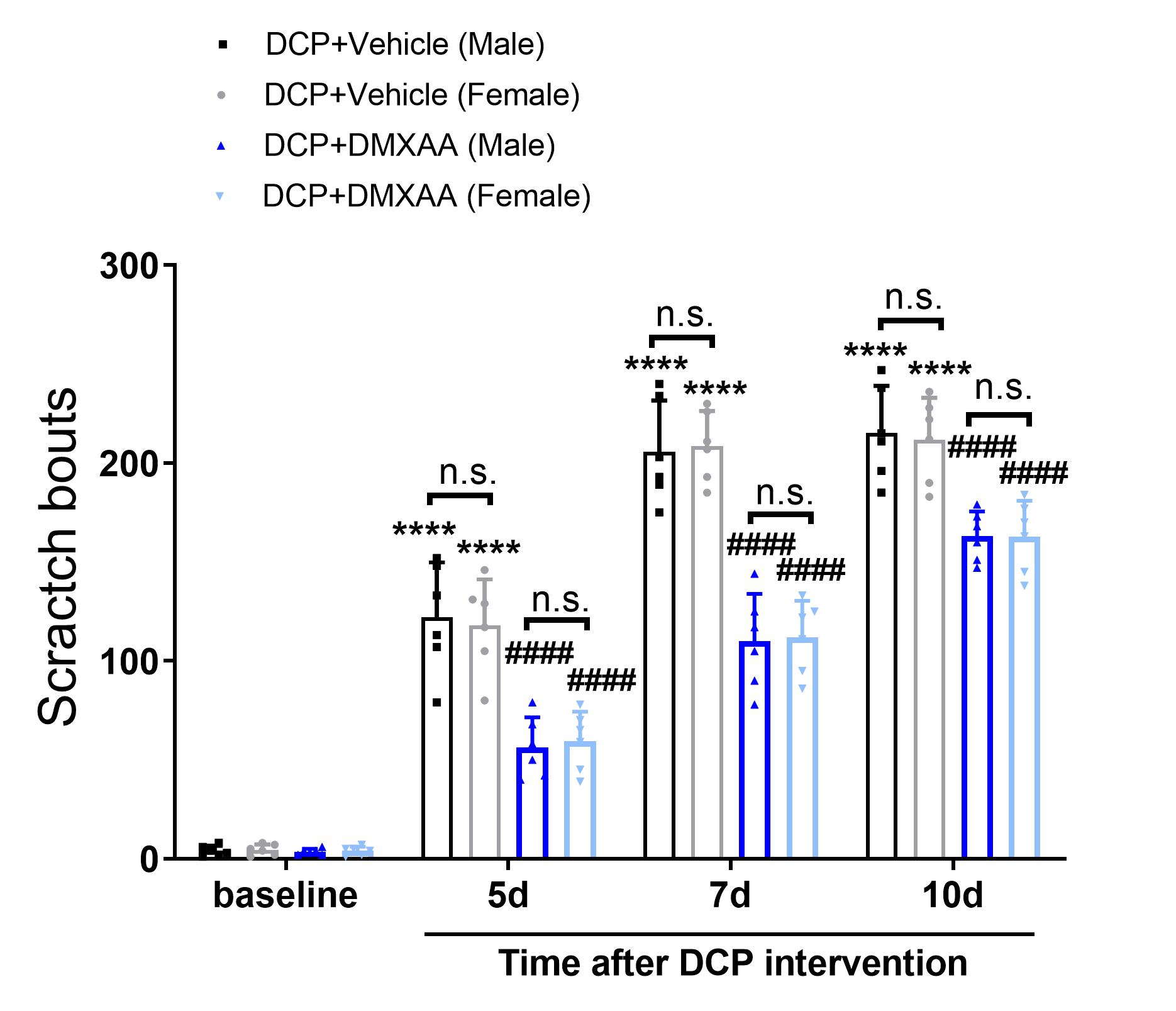

Supplement: Supplementary file 3 — Additional file 3: Figure S3. The murine STING agonist DMXAA reduces contact dermatitis-induced pruritus in both male and female mice. Mice received two injections of DMXAA daily from day 3 to 4 after DCP intervention. DCP-induced chronic scratching behaviors in male and female mice are equally alleviated by DMXAA treatment. Compared with baseline, ****P < 0.0001. Compared with group DCP + Vehicle, ####P < 0.0001. n = 6 mice/group. Data are expressed as mean ± SD and analyzed by two-way ANOVA with Bonferroni post hoc test. n.s., not significant. [file 12974_2023_2783_MOESM3_ESM.jpg]
